# LSM1-mediated Major Satellite RNA decay is required for nonequilibrium histone H3.3 incorporation into parental pronuclei

**DOI:** 10.1038/s41467-023-36584-z

**Published:** 2023-02-21

**Authors:** Jiang Zhu, Kang Chen, Yu H. Sun, Wen Ye, Juntao Liu, Dandan Zhang, Nan Su, Li Wu, Xiaochen Kou, Yanhong Zhao, Hong Wang, Shaorong Gao, Lan Kang

**Affiliations:** 1 Institute for Regenerative Medicine, Shanghai East Hospital, Shanghai Key Laboratory of Signaling and Disease Research, School of Life Sciences and Technology, Tongji University, 200120 Shanghai, China; 2 Frontier Science Center for Stem Cell Research, Tongji University, 200092 Shanghai, China; 3 Institute of Biophysics, Chinese Academy of Sciences, 100101 Beijing, China; 4 University of Chinese Academy of Sciences, 100049 Beijing, China; 5 Departments of Biology, University of Rochester, 14642 Rochester, NY USA; 6 Clinical and Translation Research Center of Shanghai First Maternity & Infant Hospital, School of Life Sciences and Technology, Tongji University, 200092 Shanghai, China

**Keywords:** Embryology, Reprogramming

## Abstract

Epigenetic reprogramming of the parental genome is essential for zygotic genome activation and subsequent embryo development in mammals. Asymmetric incorporation of histone H3 variants into the parental genome has been observed previously, but the underlying mechanism remains elusive. In this study, we discover that RNA-binding protein LSM1-mediated major satellite RNA decay plays a central role in the preferential incorporation of histone variant H3.3 into the male pronucleus. Knockdown of Lsm1 disrupts nonequilibrium pronucleus histone incorporation and asymmetric H3K9me3 modification. Subsequently, we find that LSM1 mainly targets major satellite repeat RNA (MajSat RNA) for decay and that accumulated MajSat RNA in Lsm1-depleted oocytes leads to abnormal incorporation of H3.1 into the male pronucleus. Knockdown of MajSat RNA reverses the anomalous histone incorporation and modifications in Lsm1-knockdown zygotes. Our study therefore reveals that accurate histone variant incorporation and incidental modifications in parental pronuclei are specified by LSM1-dependent pericentromeric RNA decay.

## Abstract (web summary)

Asymmetric histone modifications in parental pronuclei displays epigenetic regulation. Here the authors reveal the role of LSM1-mediated Major Satellite RNA decay in the H3 variant incorporation and modifications in male pronucleus.

## Introduction

The development of a multicellular organism with its array of organs and tissues is a systematic process in which cells acquire different identities with exquisite spatiotemporal precision through mechanisms called cell fate decisions^1^. This process is implemented by gene regulatory networks, of which epigenetic regulation is a well-studied and critical part^2^. The mechanisms of epigenetic regulation and cell fate decisions have been studied in different organisms and biological processes, and the drastic epigenetic changes in mouse early embryos make them excellent models for conducting mechanistic research^3^. Mammalian life starts with fertilization, the process by which sperm and oocytes come together to establish a totipotent zygote. Along with great changes in cell fate, epigenetic profiles undergo dramatic fluctuations^4^. More importantly, the epigenetic changes in the early embryo do not simply occur at the overall level but are under precise control, especially at the zygote stage.

After fertilization, the oocyte extrudes the second polar body, and the remaining maternal genome exists in a nucleosomal configuration, while the paternal genome from sperm experiences protamine-to-histone exchange. The genomes remain separated as male and female pronuclei before being reunified during the M phase in late zygotes^5,6^. Although they reside in the same cytoplasm, the male and female pronuclei are heterogeneous in many ways. In addition to asynchronous DNA replication and transcriptional activity, they exhibit asymmetric epigenetic states, especially DNA methylation and histone modification^7^. Genome-wide DNA demethylation occurs mainly in the male pronucleus^8,9^, while H3K9me2 and H3K9me3 modifications exclusively reside in the female pronucleus^10,11^. These accurate epigenetic modifications safeguard normal development of the early embryo. Our previous study illustrated the profile and importance of H3K9me3 in mouse embryo development^12^. Disturbances in Suv39h1 and Suv39h2 impair the onset of development^11,13^. However, how asymmetric H3K9me3 is first established and maintained in mammalian zygotes remains unknown.

In fact, in addition to histone modifications, the histone itself exhibits a pronucleus-specific pattern. Mammalian cells express three major types of histone H3 variants, H3.1, H3.2, and H3.3, and their sequences are very conserved. H3.3 differs from H3.1/H3.2 counterparts in only four or five amino acid residues^14,15^. After fertilization, H3.3 is preferentially and rapidly incorporated into the male pronucleus, taking the place of protamines, whereas H3.1 and H3.2 are found in both pronuclei but are present at higher levels in the female pronucleus than in the male pronucleus^16–18^. H3.3 is important for early embryo development, and depletion of H3.3 compromises normal preimplantation development^19^. H3.3 incorporation is mediated by HIRA or DAXX–ATRX complexes in a cell cycle-independent manner^20,21^. In contrast, H3.1/H3.2 incorporates into chromatin in a replication-coupled manner with the help of the CAF-1 histone chaperone complex^22^. Interfering with HIRA leads to embryo arrest at the zygote stage due to abnormal H3.3 incorporation^23^, and loss of DAXX and ATRX results in extensive apoptosis and trophoblast development failure owing to disturbance of H3.3 or H3.1/H3.2 incorporation^24,25^. Recently, low input ChIP-seq analysis of H3.3 delineates its landscape in early embryo development and highlights the timely transition from non-canonical H3.3 pattern toward a canonical pattern supports the developmental program of early embryos^26^. In somatic cells, H3.3 accumulates modifications related to gene activation, including methylation at K4, K36, and K79 and acetylation at K9 and K14, and is deprived of suppressive modifications, including K9 and K27 methylation^27^. Biochemistry studies have also shown that H3 variants display different tendencies toward histone posttranslational modifications, for example, H3K9me3 modification is more enriched at H3.1 than at H3.3^28^. The asymmetric H3 variant incorporation between pronuclei and its effects on epigenetic modification establishment in zygotes are far from illuminated. Furthermore, elucidating the mechanism of the establishment and maintenance of nonequilibrium pronuclear histones and modifications may shed light on precise and locus-specific epigenetic modifications.

LSM1 is an RNA-binding protein with an Sm-like domain; it usually exists as an Lsm1–7-Pat1 complex and serves as a key factor in the RNA decay pathway^29^. In this study, we found that germ cell-derived Lsm1 participates in nonequilibrium H3.3 incorporation and asymmetric H3K9me3 establishment in mouse zygotes, which is accomplished through MajSat RNA decay.

## Results

### Nonequilibrium pronuclear H3K9me3 is specified by LSM1

**Fig. 1:**
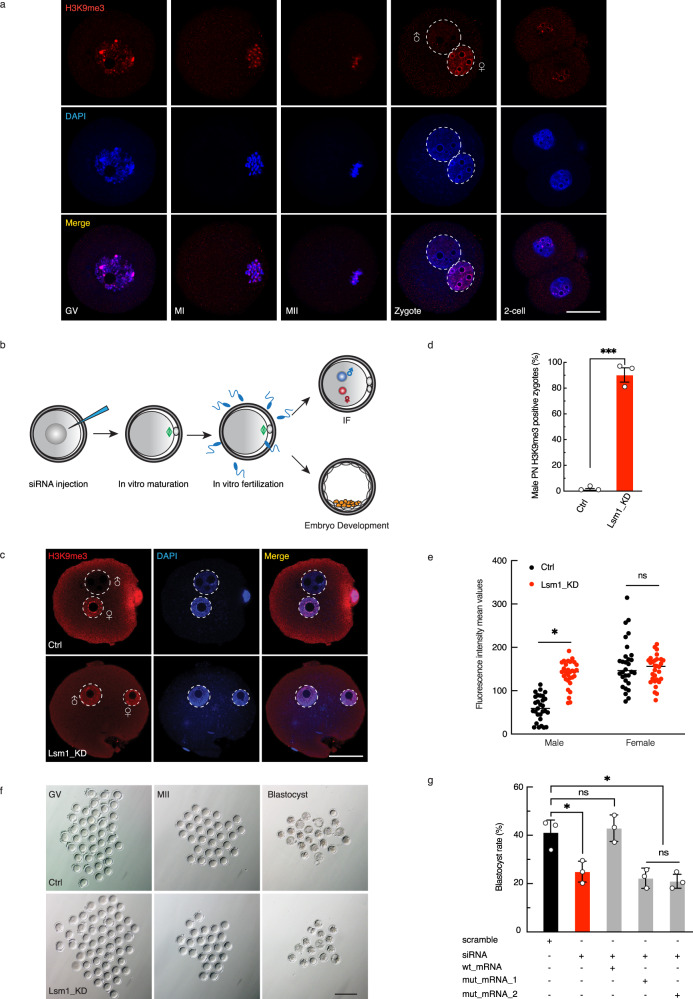
Nonequilibrium pronuclear H3K9me3 is specified by LSM1. **a** Immunostaining for H3K9me3 in nuclei from GV-, MI-, and MII-stage oocytes and 1-cell and 2-cell embryos. Scale bars, 20 µm. Representative images from 3 biological replicates, with each replicate containing ~10 oocytes/embryos. **b** Scheme for the Lsm1 oocyte-stage KD experiment. Lsm1 siRNA was injected at the GV stage after which in vitro maturation (IVM) and in vitro fertilization (IVF) were performed. Some of the fertilized oocytes were fixed at the zygote stage for immunostaining, and the others were cultured in vitro for embryo development assays. **c** Immunostaining for H3K9me3 in representative pronucleus of control (Ctrl) and Lsm1 knockdown (Lsm1_KD) zygotes. Scale bars, 20 µm. Representative images from 3 biological replicates, with each replicate containing ~10 zygotes. **d** Bar graph showing percentage of H3K9me3 positive male PN zygote in different groups. The statistical data are expressed as mean ± SEM, ***p < 0.001 (*p* = 0.0004) by two-sided Student’s *t* test for each comparison. Each treatment contains 3 biological replicates, with each replicate containing ~30 zygotes. **e** Scatterplot showing H3K9me3 fluorescence intensity mean values in pronucleus of Ctrl and Lsm1_KD groups. The statistical data are expressed as mean ± SEM, *p < 0.05 (*p* = 0.0289) and ns (*p* = 0.7834) means not significant by two-sided Student’s *t* test for each comparison. Each treatment contains 3 biological replicates, with each replicate containing ~10 zygotes. **f** Representative images of injected GV-stage oocytes, mature oocytes and blastocysts in the Ctrl and Lsm1_KD groups. Scale bars, 100 µm. Representative images from 3 biological replicates, with each replicate containing ~35 injected GV-oocytes. **g** Quantification of embryo development competence in response to Lsm1 KD with or without different versions of Lsm1 RNA. The statistical data are expressed as mean ± SEM, *p < 0.05 and ns means not significant by one-way ANOVA test. Each treatment contains 3 biological replicates, with each replicate containing ~35 embryos.

Histone modification fluctuates and is precisely controlled during early embryo development, and aberrant modification leads to developmental failure^30^. Upon fertilization, protamines are replaced by histones in the male pronucleus, and histone modifications are gradually established in a model different from that in the female pronucleus. This asymmetric pronuclear histone pattern is usually persistent until after the 2-cell stage, with asymmetric H3K9me3 as a typical example^10,11,31^. H3K9me3 was distributed in the whole nucleus during oocyte maturation and was mainly restricted to the female pronucleus after fertilization (Fig. 1a). When we explored the function of zygote-enriched factors from our previous proteomic data^32^, we fortuitously found a vital role of LSM1 in the asymmetric pattern of H3K9me3. Then, we realized that this might lead us to unveil the unclear mechanism of nonequilibrium pronuclear histone establishment.

In a functional examination, Lsm1 siRNA was microinjected into GV-stage oocytes, after which in vitro maturation and fertilization were performed. Then, the embryos were used to detect histone epigenetic modifications, together with embryo development analysis (Fig. 1b). The siRNA produced about 45% and 60% depletion of Lsm1 mRNA in MII oocytes and zygotes respectively (Supplementary Fig. 1a, b). Lsm1 knockdown (KD) leaded to the remarkable disappearance of asymmetric H3K9me3, in more than 90% of the zygotes, H3K9me3 in male pronucleus was significantly increased (Fig. 1c–e). That is, the male pronucleus, which was supposed to preclude H3K9me3, obtained this modification after Lsm1 KD.

With the dramatically abnormal epigenetic profiles, we sought to determine whether Lsm1 KD could cause developmental defects. When we traced embryo development, the blastocyst formation rate in the KD group decreased to 25%, whereas it was 41% in the control group (Fig. 1f, g). The decreased embryo development competence, blastocyst rate, could be rescued by co-injected Lsm1 mRNA, which possessed synonymous mutation on the siRNA binding site, but not by the two mutant mRNAs with impaired Sm-fold domain^33–36^ (Fig. 1g). Even before fertilization, Lsm1 KD caused a severe defect—abnormal spindle orientation—and only half of the oocytes could accomplish first polar body extrusion and maturation in the KD group compared with the control group (Fig. 1f and Supplementary Fig. 2a–d). This phenomenon seemed unrelated with H3K9me3 (Supplementary Fig. 2e). It seemed that Lsm1 functioned very early, so we also performed zygote siRNA injection to clarify the functional time window of Lsm1 (Supplementary Fig. 1c). Indeed, zygote injection of either Lsm1 siRNA or scramble siRNA did not affected the asymmetric H3K9me3 (Supplementary Fig. 1d, e) or the embryo development (Supplementary Fig. 1f–h). These observations strongly suggested LSM1 as a vital factor contributing to nonequilibrium pronuclear H3K9me3 and early embryo development.

### LSM1 modulates the pronucleus-specific localization of HP1β, ATRX, H3.1/3.2 and H3.3

**Fig. 2:**
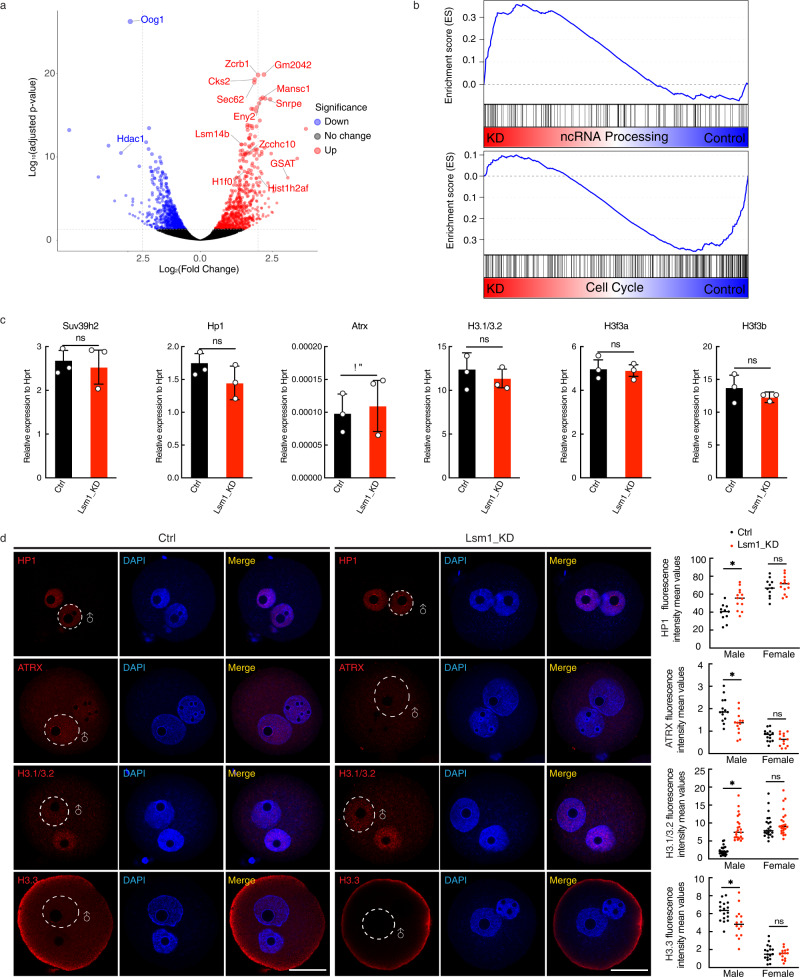
LSM1 modulates the pronucleus-specific localization of HP1β, ATRX, H3.1/3.2, and H3.3. **a** Volcano plot showing differentially expressed genes between Ctrl and Lsm1_KD zygotes. Red dots represent upregulated genes in Lsm1_KD zygotes compared with control zygotes, and blue dots represent downregulated genes. Adjusted *p*-values (two-sided) attained by the Wald test are corrected for multiple testing using the Benjamini and Hochberg method by default (*n* = 2 biologically independent samples). **b** GSEA plot showing preferential upregulation of the ncRNA processing pathway and downregulation of the cell cycle processing pathway following Lsm1 KD. **c** qPCR validation of H3K9me3 modification-related genes and H3 genes in Lsm1_KD zygotes. The statistical data are expressed as mean ± SEM, ns means not significant by two-sided Student’s *t* test for each comparison, Suv39h2 (*p* = 0.7478), Hp1β (*p* = 0.3525), Atrx (*p* = 0.8270), H3.1/3.2 (*p* = 0.6474), H3f3a (*p* = 0.8779), H3f3b (*p* = 0.5152). Each reaction for qPCR analysis contains 3 biological replicates, with each replicate from 50 zygotes. **d** Immunostaining for HP1β, ATRX, H3.1/3.2, and H3.3 in representative pronucleus and fluorescence intensity analysis of Ctrl and Lsm1_KD zygotes. Scale bars, 20 µm. Representative images from 3 biological replicates, with each replicate containing at least 5 zygotes. The statistical data are expressed as mean ± SEM, *p < 0.05 and ns means not significant by two-sided Student’s *t* test for each comparison, HP1β: male *p* = 0.0340, female *p* = 0.5630, ATRX: male *p* = 0.0410, female *p* = 0.4592, H3.1/3.2: male *p* = 0.0128, female *p* = 0.7540 and H3.3: male *p* = 0.0394, female *p* = 0.8779. Each treatment contains 3 biological replicates, with each replicate containing at least 5 zygotes.

The two phenomenon - aberrant equilibrium of H3K9me3 and impaired embryo development - caused by Lsm1 KD prompted us to search for the underlying factors. We performed total RNA sequencing (total RNA-seq) using embryos at the zygote stage, which revealed that the transcriptional profile of the Lsm1_KD zygotes was reproducibly distinct from that of the controls (Supplementary Fig. 3a). Among the differentially expressed genes induced by Lsm1 KD, the upregulated transcripts (1538, log_2_ fold change [FC] > 1, false discovery rate [FDR] < 0.05) were much more abundant than the decreased ones (638) (Fig. 2a). This result is reasonable given the known RNA decay function of LSM1^37,38^. Gene Ontology (GO) analysis and further GSEA analysis showed that the differentially expressed protein-coding genes were enriched for mRNA processing and RNA splicing, which coincided with LSM1’s RNA-binding function, and for terms related to the cell cycle and chromosome segregation, which would explain the embryo development defect (Fig. 2b and Supplementary Fig. 3b, c).

To find out potential reasons for the abnormal H3K9me3 observed, we explored genes related to H3K9me3 establishment and histone H3 variants believed to be different between pronuclei, such as Suv39h2, Hp1β, Atrx, Daxx, H3f3a, and H3f3b, and found that they rarely changed between the Lsm1_KD and control groups in our RNA-seq data (Supplementary Fig. 3d). These sequencing results were further confirmed by qPCR (Fig. 2c). However, this finding is reasonable because the precise control of histone modification should not be regulated at the transcriptional level for these universal factors. Therefore, we sought to determine the protein levels of these factors. As the immunostaining showed, HP1β, a key protein participating in heterochromatin establishment and maintenance^39,40^, became more condensed at the male pronucleus after interference with Lsm1, coinciding with the increased H3K9me3. On the other hand, the histone H3.3 lost its male pronucleus location, together with ATRX, which plays a key role in the incorporation of H3.3^20^. H3.1/3.2, taking the place of H3.3, became more enriched in the male pronucleus (Fig. 2d).

Together, these data suggest that the aberrant equilibrium H3K9me3 caused by Lsm1 KD might be a result of the incorrect localization of the enzymes and cofactors. The simultaneous change of H3.3 to H3.1 even implied that disordered incorporation of histone variants occurred upstream of the aberrant equilibrium H3K9me3.

### LSM1 interacts with major satellite RNA and regulates its decay

**Fig. 3:**
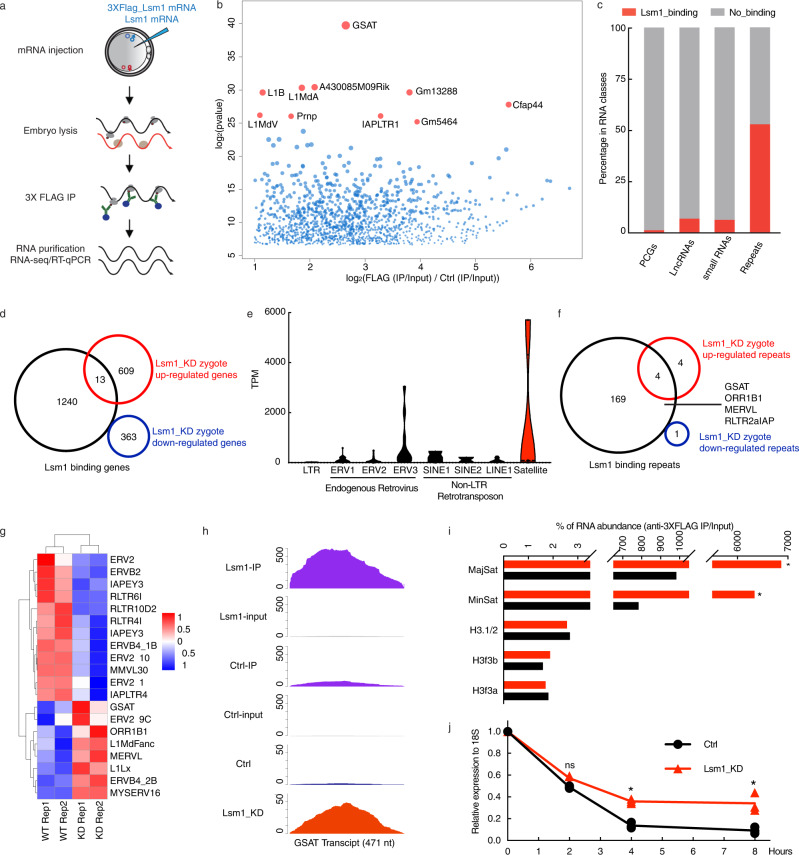
LSM1 interacts with major satellite RNA and regulates its decay. **a** Scheme for the LSM1 RIP-seq and RIP-qPCR experiments. **b** Volcano plot showing LSM1-binding RNAs in zygotes. The red dots represent the top 10 targets of LSM1 in zygotes. *p*-values (two-sided) attained by the Wald test are corrected for multiple testing using the Benjamini and Hochberg method by default (*n* = 2 biologically independent samples). **c** Bar graph showing the LSM1 binding ratios in different RNA classes. **d** Venn diagrams showing the overlap of LSM1-binding protein-coding genes and up-/downregulated genes in Lsm1_KD zygotes. **e** Violin plot showing the distribution of LSM1-binding reads in different repeat families. **f** Venn diagrams showing the overlap of LSM1-binding repeats and up-/downregulated repeats in Lsm1_KD zygotes. **g** Heatmap showing differentially expressed repeats between Ctrl and Lsm1_KD zygotes. **h** Genome browser tracks showing LSM1 RIP-seq peaks and Lsm1_KD zygote RNA-seq peaks at the MajSat locus. **i** RIP-qPCR validation of target binding by LSM1. The statistical data are expressed as mean ± SEM, *p < 0.05 (MajSat: *p* = 0.0182, MinSat: *p* = 0.0228) by two-sided Student’s *t* test for each comparison. Each reaction for RIP-qPCR analysis contains 3 biological replicates, with each replicate from 300 zygotes. **j** Measurement of MajSat RNA decay in Ctrl and Lsm1_KD zygotes. Samples were collected following transcriptional inhibition using actinomycin D (ActD) for the indicated time. MajSat RNA amounts were measured by qPCR according to 18S RNA. The statistical data are expressed as mean ± SEM, *p < 0.05 (*p* = 0.0189 for 4 hours and *p* = 0.0112 for 8 h) and ns (*p* = 0.8217 for 2 h) means not significant by two-sided Student’s *t* test for each comparison. Each reaction for qPCR contains 3 biological replicates, with each replicate from ~50 zygotes.

Then, we traced the clues regarding LSM1’s action to clarify the mechanism of the establishment and maintenance of pronuclear histone nonequilibrium. Considering that LSM1 is an RNA-binding protein, we decided to examine its direct binding RNA using RNA immunoprecipitation sequencing (RIP-seq). As there is no effective commercial antibody for LSM1 immunoprecipitation, we employed FLAG-tagged LSM1 overexpression (OE) instead. In vitro-transcribed 3XFlag-tagged Lsm1 mRNA was injected into early zygotes, and after 5 hours of culture, late zygotes were collected for FLAG immunoprecipitation and total RNA library construction and sequencing (Fig. 3a). Using Lsm1 mRNA-injected zygotes as negative controls, we identified 1433 transcripts that directly interacted with LSM1 (Fig. 3b). Among these transcripts, 70%, 12%, 17% and 1% were protein-coding genes, repeat elements, lncRNAs and small ncRNAs, respectively (Supplementary Fig. 4a). However, when we calculated the LSM1 binding elements percentage in zygote expressed elements (TPM > 1) for their corresponding families, surprisingly, repeat elements presented the highest percentage, which suggested that LSM1 had a high binding preference for repeat elements in zygotes (Fig. 3c).

Since protein-coding genes and repeat elements were dominant classes in terms of transcript number and binding intensity, we proceeded to mine the RIP-seq data for these two classes. GO analysis of these LSM1-binding protein-coding genes showed that they were related to molecular transport and immune cell activation, showing lower correlation with embryo development (Supplementary Fig. 4b). Combined with our previous total RNA-seq data on Lsm1_KD zygotes, the data revealed that only 13 upregulated protein-coding genes were directly bound by LSM1, while none of the downregulated genes showed LSM1-binding properties (Fig. 3d). These results implied that LSM1 regulates protein-coding genes mainly in an indirect way. Furthermore, the absence of Hp1β, Atrx, H3.1, H3.2, and H3.3 in the LSM1-binding list in turn supported our previous deduction that these molecules may not be direct targets of LSM1.

Given that repeat elements can be classified into many subfamilies, we further calculated the percentage of LSM1 binding elements to its expressed ones for each subfamily and found that the satellite subfamily had the highest contribution (Supplementary Fig. 4c). The transcripts per kilobase million (TPM) value visualized by the violin plot made it clear that the satellite subfamily was the top subfamily (Fig. 3e). Combined with the total RNA-seq data of Lsm1_KD zygotes, the data remarkably revealed that 4 upregulated repeats, GSAT, ORR1B1, MERVL, and RLTR2aIAP, were directly bound by LSM1 (Fig. 3f). This result prompted us to focus on GSAT, which is normally called major satellite (MajSat) RNA. It was very noticeable in the volcano plot (Fig. 3b) and presented as the most highly unregulated repeat element upon Lsm1 KD in heat map (Fig. 3g), which was also confirmed by qPCR (Supplementary Fig. 4d). So we further assessed the peaks in the RIP-seq results, which confirmed the strong interaction between LSM1 and MajSat RNA (Fig. 3h). RIP-qPCR further confirmed the strong interaction of MajSat RNA and LSM1. MajSat RNA was ~10-fold enriched in the LSM1 RIP samples compared with the controls, whereas the levels of H3.1/3.2, H3f3a, and H3f3b, which maintained the same expression in Lsm1_KD zygotes, were comparable between groups (Fig. 3i).

Considering that LSM1 is known to participate in RNA decay^29^, we performed an RNA decay assay to examine whether LSM1 induced MajSat RNA decay. Consistent with our hypothesis, the decay of MajSat RNA was obviously mitigated in Lsm1_KD zygotes (Fig. 3j). Moreover, a negative correlation pattern was found: MajSat RNA was downregulated from MII oocytes to zygotes (Supplementary Fig. 4e), whereas the opposite result was observed for Lsm1 (Supplementary Fig. 4f). These data suggest MajSat RNA as one of the most important decay targets of LSM1 in mouse zygotes.

### Major satellite RNA mediates the impact of Lsm1 KD on nonequilibrium pronuclear H3K9me3 and early embryo development

**Fig. 4:**
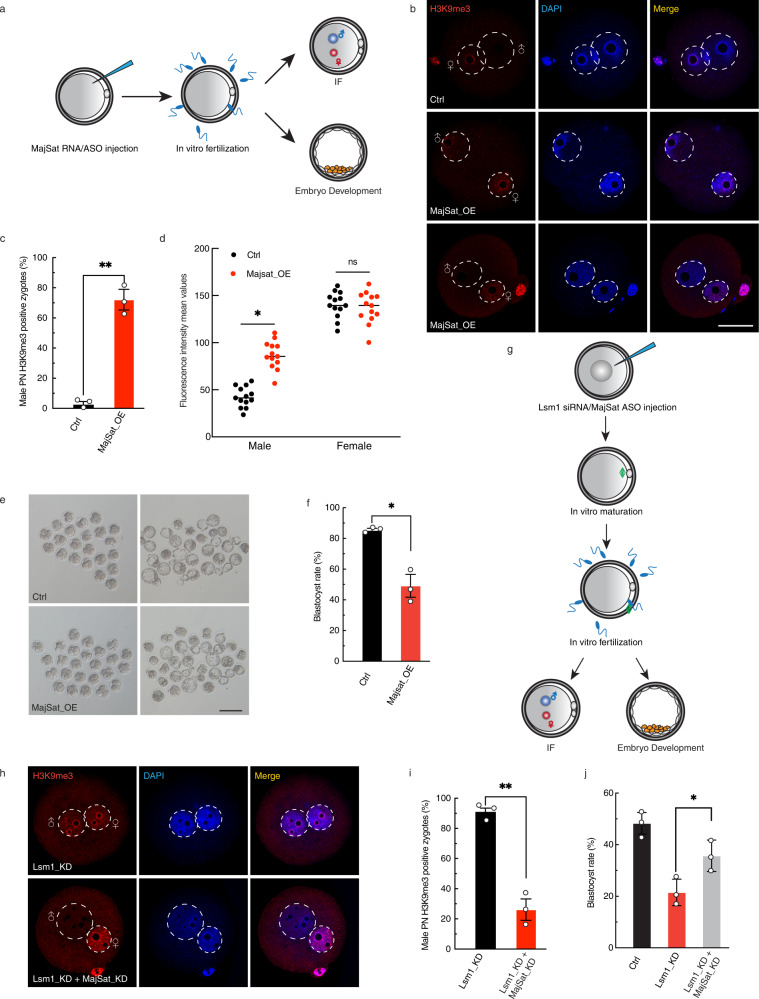
Major satellite RNA mediates the impact of Lsm1 KD on nonequilibrium pronuclear H3K9me3 and early embryo development. **a** Scheme for MajSat oocyte-stage OE and KD experiments. In vitro-transcribed MajSat RNA or ASOs were injected into MII oocytes. After IVF, some of the fertilized oocytes were fixed at the zygote stage for H3K9me3 immunostaining, and the others were cultured in vitro for embryo development assays. **b** Immunostaining for H3K9me3 in representative pronucleus of Ctrl and major satellite RNA overexpression (MajSat_OE) zygotes. Scale bars, 20 µm. Representative images from 3 biological replicates, with each replicate containing ~30 zygotes. **c** Statistics for male pronucleus H3K9me3 in Ctrl and MajSat_OE zygotes. The statistical data are expressed as mean ± SEM, **p < 0.01 (*p* = 0.0016) by two-sided Student’s *t* test for each comparison. Each treatment contains 3 biological replicates, with each replicate containing ~30 zygotes. **d** Scatterplot showing H3K9me3 fluorescence intensity mean values in pronucleus of Ctrl and MajSat_OE zygotes. The statistical data are expressed as mean ± SEM, *p < 0.05 (*p* = 0.0158) and ns (*p* = 0.8390) means not significant by two-sided Student’s t test for each comparison. Each treatment contains 3 biological replicates, with each replicate containing at least 5 zygotes. **e**, **f** Quantification of embryo development competence in response to major satellite RNA. Images of 2-cell embryos and blastocysts (**e**) and statistics for blastocyst rates (**f**) for the Ctrl and MajSat_OE groups were represented. Representative images from 3 biological replicates, with each replicate originating from ~35 injected oocytes. The statistical data are expressed as mean ± SEM, *p < 0.05 (*p* = 0.0119) by two-sided Student’s *t* test for each comparison. Each treatment contains 3 biological replicates, with each replicate originating from ~35 injected oocytes. **g** Scheme for rescue experiments. Lsm1 siRNA and MajSat ASOs were injected at the GV stage, after which IVM and IVF were performed. Some fertilized oocytes were fixed at the zygote stage for H3K9me3 immunostaining, and the others were cultured in vitro for embryo development assays. **h**, **i** Immunostaining of H3K9me3 in Lsm1_KD and Lsm1_KD + MajSat_KD zygotes. Images of H3K9me3 (**h**) and fluorescence intensity mean values (**i**) in pronucleus of Lsm1_KD and Lsm1_KD + MajSat_KD zygotes were represented. Scale bars, 20 µm. Representative images from 3 biological replicates, with each replicate originating from ~30 zygotes. The statistical data are expressed as mean ± SEM, **p < 0.01 (*p* = 0.0016) by two-sided Student’s *t* test for each comparison. Each treatment contains 3 biological replicates, with each replicate originating from ~30 zygotes. **j** Statistics for the blastocyst rates of Ctrl, Lsm1_KD and Lsm1_KD + MajSat_KD zygotes. The statistical data are expressed as mean ± SEM, *p < 0.05 (*p* = 0.0285) by one-way ANOVA test. Each treatment contains 3 biological replicates, with each replicate originating from ~35 injected GV-oocytes.

The above findings raised the question of whether MajSat RNA was the downstream factor of LSM1 with regard to the nonequilibrium pronuclear H3K9me3 and following embryo development. To answer this question, we carried out OE and KD of MajSat RNA (Fig. 4a). Considering that MajSat RNA decreased by half from the MII stage to the zygote stage, we decided to conduct injections at the MII stage. Overexpression of MajSat RNA was carried out by injection of in vitro-transcribed forward-strand RNA with overexpression efficiency confirmed qPCR (Supplementary Fig. 5a). Consistent with our expectation, in MajSat OE group, H3K9me3 gained a bipronuclear distribution similar to that in Lsm1_KD zygotes, with significantly increased H3K9me3 signal in male pronucleus (Fig. 4b), but not all zygotes presented this equilibrium H3K9me3 phenomenon, with ~30% zygotes exhibited the same pattern as the control group (Fig. 4c, d). This implies that other downstream factors exist in addition to MajSat RNA. Together with abnormal H3K9me3, MajSat RNA OE resulted in a significantly decreased blastocyst rate; the rate decreased from 85% in the control group to 49% in the OE group (Fig. 4e, f). On the other hand, knockdown of MajSat RNA using antisense oligos (ASOs) (MajSat ASO: AUAUUTCACGTCCTAAAGUG) had little influence on asymmetric H3K9me3 or embryo development (Supplementary Fig. 5f–i). Furthermore, when we overexpressed MajSat RNA at the early zygote stage, H3K9me3 modification and embryo development were not affected (Supplementary Fig. 5b–e), suggesting that MajSat RNA also functions very early, similar to LSM1.

To further clarify the relationship between LSM1 and MajSat RNA, we tested whether MajSat RNA KD could rescue the phenotypes of Lsm1 KD (Fig. 4g). Under the injection of MajSat ASOs together with Lsm1 KD, asymmetric H3K9me3 was regained, and the male pronucleus began to sequester H3K9me3 (Fig. 4h), about 65% zygotes can be rescued (Fig. 4i), which again implies that other downstream factors exist in addition to MajSat RNA. Along with the amelioration of the epigenetic defect, recovery of embryo development was observed, with the blastocyst formation rate increasing from 20% to 35%, close to that of the control group (Fig. 4j and Supplementary Fig. 5j, k).

Taken together, the results indicated that overabundance of MajSat RNA caused by Lsm1 KD was one of the key reasons for the disturbance of the nonequilibrium pronuclear H3K9me3 and early embryo development.

### Incorrect H3.1/3.2 incorporation introduced by major satellite RNA

**Fig. 5:**
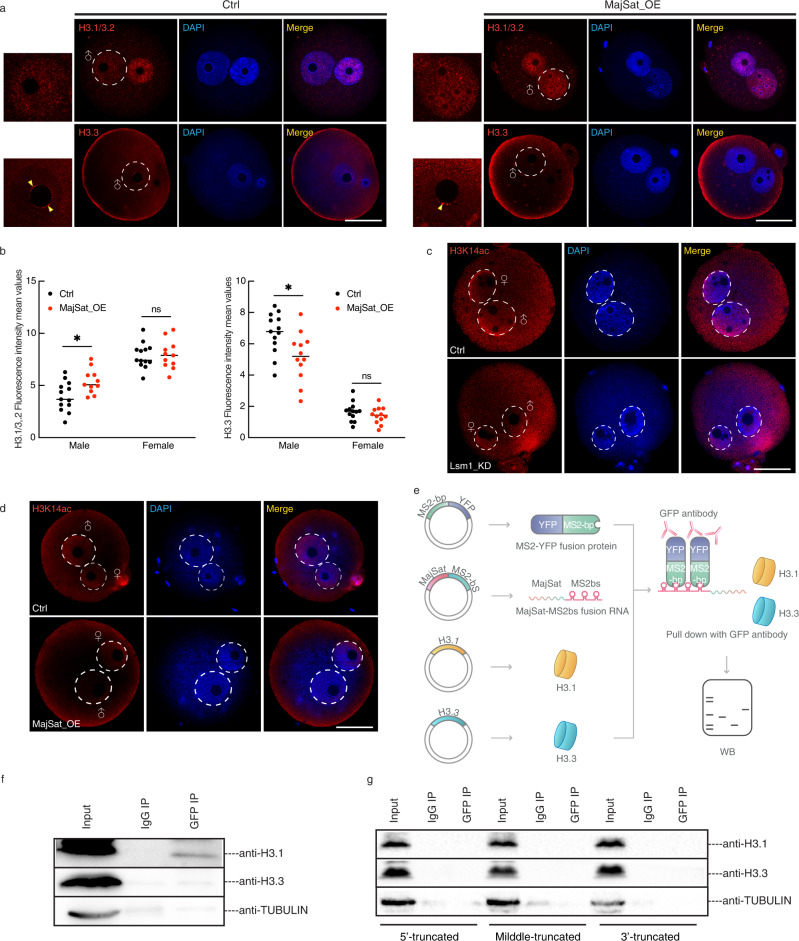
Abnormal H3K9me3 is incidental to incorrect H3.1/3.2 incorporation introduced by major satellite RNA. **a** Immunostaining for H3.1/3.2 and H3.3 in representative pronucleus of Ctrl and MajSat_OE zygotes. Scale bars, 20 µm. Representative images from 3 biological replicates, with each replicate containing at least 10 zygotes. **b** Scatterplot showing H3.1/3.2 and H3.3 fluorescence intensity mean values in pronucleus of Ctrl and MajSat_OE zygotes. The statistical data are expressed as mean ± SEM, *p < 0.05 and ns means not significant (H3.1/3.2: male *p* = 0.0438, female *p* = 0.6590, H3.3: male *p* = 0.0380, female *p* = 0.5885) by two-sided Student’s *t* test for each comparison. Each treatment contains 3 biological replicates, with each replicate containing at least 5 zygotes. **c** Immunostaining for H3K14ac in representative pronucleus of Ctrl and Lsm1_KD zygote. Scale bars, 20 µm. Representative images from 3 biological replicates, with each replicate containing at least 10 zygotes. **d** Immunostaining for H3K14ac in representative pronucleus of Ctrl and MajSat_OE zygote. Scale bars, 20 µm. Representative images from 3 biological replicates, with each replicate containing at least 10 zygotes. **e** Scheme for the RNA pulldown to determine the interaction between MajSat RNA and H3.1/H3.3. **f**, **g** Pulldown of H3.1, H3.3 using intact and truncated MajSat RNA. Representative images from 3 biological replicates.

Previously, we found that precise localization of H3.1/3.2 and H3.3 together with H3K9me3 was impaired by Lsm1 KD. As MajSat RNA is a key direct effector of LSM1, overexpression of MajSat RNA could indeed reproduce the same phenomenon. H3.3, which is believed to be located in the male pronucleus, almost disappeared from its former place after MajSat RNA OE, 63% zygote showed decreased H3.3 deposition. On the other hand, H3.1/3.2 became more condensed in the male pronucleus, 68% zygote showed increased H3.1/3.2 deposition (Fig. 5a, b). That is, overexpression of MajSat RNA also resulted in the loss of nonequilibrium pronuclear histone variants. Furthermore, knockdown of MajSat RNA partially ameliorated the abnormal H3.1/3.2 and H3.3 male pronuclear distribution in Lsm1_KD zygotes (Supplementary Fig. 6a).

The synchronous changes in H3K9me3 and histone H3 variants prompted the hypothesis that incorrect H3.1/3.2 incorporation resulted in abnormal H3K9me3. As described previously, H3.1/3.2 and H3.3 exhibit different modification preferences; in particular, K9me3 and K27me3 are more enriched on H3.1, while K14ac is more common on H3.3^28^. Therefore, if the H3K9me3 modification change is a consequent event of H3 variant incorporation, the same should happen to H3K14ac and H3K27me3. As expected, the H3K14ac signal in the male pronucleus decreased sharply under Lsm1 KD (Fig. 5c). The same phenomenon was found in MajSat RNA-overexpressing zygotes, and H3K14ac lost the male pronuclear distribution, similar to the case in Lsm1_KD zygotes (Fig. 5d). However, H3K27me3 was an exception, after Lsm1 KD, H3K27me3 still exhibited a female pronucleus-specific distribution (Supplementary Fig. 6b). This could be explained by a shortage of EZH2, the main factor of PRC2, as it accumulates from the late zygote stage^11,41,42^.

The next question was how MajSat RNA mediated this process. Previous studies have reported the chromatin association property of MajSat RNA^43^, moreover, it is also located in the male pronucleus^43^, so we tested the possibility that MajSat RNA might possess different affinities for H3.1 and H3.3. To detect the binding between RNA and protein, we employed the MS2 system^44^, MS2 stem-loop-linked MajSat RNA, GFP-tagged MS2 protein, and mouse H3.1 and H3.3 were all introduced into HEK293T cells (Fig. 5e). The IP efficiency was confirmed by MajSat RNA qPCR, showing ~10 times enrichment compared to the IgG control (Supplementary Fig. 6c). When detecting histones by western blot analysis, we found that H3.1 was pulled down by MajSat RNA, while H3.3 was almost undetectable (Fig. 5f). To further confirm the interaction domain between MajSat RNA and H3.1, pull-down experiment was conducted using the RNA with truncation at its 5’-end, middle and 3′-end, the results showed that none of part was dispensable for the MajSat RNA’s H3.1 binding ability (Fig. 5g). Thus, we supposed that H3.1 was recruited and incorporated into the male pronucleus by overexpressed MajSat RNA, resulting in abnormal histone modifications.

### Lsm1 originates from both gametes to help establish totipotency

**Fig. 6:**
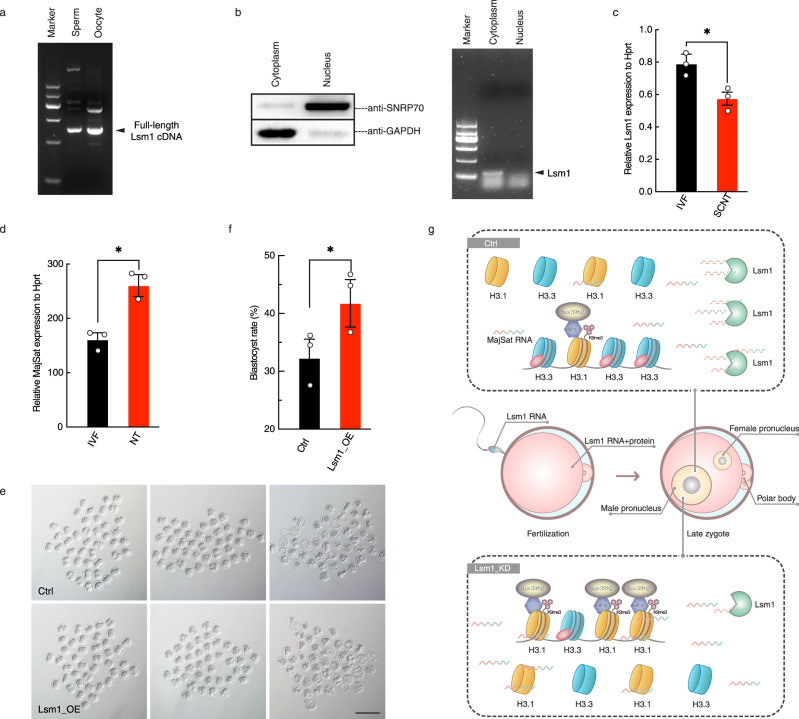
Lsm1 originates from both gametes and helps establish totipotency. **a** Agarose gel electrophoresis of RT–PCR amplification of full-length Lsm1 mRNA from sperm and oocytes. Representative images from 3 biological replicates. **b** Agarose gel electrophoresis of RT–PCR detection of Lsm1 mRNA from cytoplasm and nucleus of somatic cells. Representative images from 3 biological replicates. **c** Lsm1 mRNA levels between IVF and SCNT zygotes as quantified by qPCR. The statistical data are expressed as mean ± SEM, *p < 0.05 (*p* = 0.0409) by two-sided Student’s *t* test for each comparison. Each reaction for qPCR analysis contains 3 biological replicates, with each replicate from 50 zygotes. **d** MajSat RNA level between IVF and SCNT zygotes as quantified by qPCR. The statistical data are expressed as mean ± SEM, *p < 0.05 (*p* = 0.0137) by two-sided Student’s *t* test for each comparison. Each reaction for qPCR analysis contains 3 biological replicates, with each replicate from 50 zygotes. **e**, **f** Quantification of SCNT embryo developmental competence in response to Lsm1 overexpression (Lsm1_OE). Images of 2-cell embryos and blastocysts for the Ctrl and Lsm1_OE groups (**e**) and statistics for blastocyst rates (**f**) were represented. Scale bars, 100 µm. Representative images from 3 biological replicates, with each replicate originating from ~35 embryos. The statistical data are expressed as mean ± SEM, *p < 0.05 (*p* = 0.0428) by two-sided Student’s *t* test for each comparison. Each treatment contains 3 biological replicates, with each replicate contains ~35 embryos. **g** Proposed model for the regulation of nonequilibrium pronuclear histone variants and concomitant asymmetric histone modifications by LSM1-induced major satellite RNA decay.

Thus far, our results revealed that Lsm1 was indispensable for supporting nonequilibrium pronuclear histones and subsequent embryo development, mainly because it decayed MajSat RNA. Considering its functional time window, Lsm1 seemed to be a maternal factor. However, when we examined its RNA level, we found an ~1-fold increase from MII oocytes to zygotes (Supplementary Fig. 4f). This phenomenon raised the question of the origin of the additional Lsm1 mRNA. We hypothesized that Lsm1 mRNA was either transcribed immediately after fertilization or carried with the sperm. To test the first possibility, we treated zygotes with the transcription inhibitor actinomycin D, but the Lsm1 RNA level remained the same as that of the control group (Supplementary Fig. 6d). For the latter, we examined Lsm1 mRNA in sperm and found that it was indeed present (Supplementary Fig. 6e). Considering that sperm harbor many RNA fragments^45,46^, we further confirmed the existence of intact Lsm1 mRNA by both PCR (Fig. 6a) and sequencing (Supplementary Fig. 6f). Furthermore, this result was also supported by third-generation RNA-seq data of mouse sperm^47^. Thus, we clarified that Lsm1 originated from both gametes.

Given the gametal origination and critical function of Lsm1, there was a chance that the materials from sperm contribute to totipotency establishment. To investigate this possibility, we employed somatic cell nuclear transfer (SCNT), in which the donor nucleus instead of sperm was injected into the oocyte, thus the insufficient germ cell factors would be one of the reasons affecting totipotency establishment in SCNT embryo. Considering most Lsm1 mRNA existed in cytoplasm rather than nuclear in somatic cells (Fig. 6b), we sought to determine whether insufficient Lsm1 is one of the factors affecting SCNT efficiency. As expected, Lsm1 mRNA levels were ~25% lower in SCNT zygotes than in IVF zygotes (Fig. 6c); in addition, an ~1-fold higher dose of MajSat RNA was found in the SCNT group than in the control group (Fig. 6d). When we injected the in vitro-transcribed Lsm1 mRNA into MII oocytes prior to SCNT, the blastocyst rate increased from 32.3 ± 3.3% to 41.8 ± 4.1% (Fig. 6e, f). Thus, the additional Lsm1 could partially compensate for the defects in SCNT embryos.

Taken together, our findings demonstrate that the germ cell-originated factor Lsm1 safeguards nonequilibrium pronuclear histones and histone modification from MajSat RNA, thus contributing to the regulation of the totipotency establishment and embryo development.

## Discussion

Accurate control of epigenetic modifications is a very important aspect of cell fate determination. In the process of totipotency establishment and early embryo development, epigenetic reprogramming takes place and must be regulated precisely, however, the mechanisms are far from elucidated due to multiple layers of regulation^48,49^. Based on the evidence presented in this study, we propose a model for accurate histone incorporation and incidental modifications specified by LSM1 through pericentromeric RNA in the male pronucleus (Fig. 6g).

Upon fertilization, orchestral histone H3 variant incorporation into the pronucleus is critical for embryo development^50^. H3.3 is preferentially incorporated into the male pronucleus shortly after fertilization, but this asymmetry disappears after two cell stage^16,17^. Moreover, the accurate H3.3 landscapes in mouse oocytes and early embryos have been delineated, which provides us a powerful resource to study H3 variants function in embryo development^26^. ATRX and DAXX have been reported to facilitate H3.3 incorporation^20,51,52^, but the reason for pronuclear specificity is still not clear. Our data show that MajSat RNA possesses a higher affinity for H3.1 than for H3.3, and given its higher enrichment in the male pronucleus than in the female pronucleus^43^, MajSat RNA might be the key to unveiling the mechanism by which nonequilibrium pronuclear histones are established.

Switching of the histone variant results in changes in histone modifications. H3.1 and H3.3 possess distinct accessibility for several modifications; for example, compared to H3.3, H3.1 more easily obtains K9me3 and K27me2/3, whereas H3.3 preferentially obtains K14ac^28^. In our study, Lsm1 KD or MajSat RNA OE led to incorrect H3.1/3.2 incorporation and synchronous changes in H3K9me3 and H3K14ac, but not in H3K27me3, implying that different precise regulations existed. H3K9me3 and H3K27me3 are regarded as two main repressive histone modifications usually marking heterochromatin, both of which exhibit female pronucleus-specific localization in zygotes. Indeed, decreased opening of chromatin indicated by the high DAPI signal could be found in the male pronucleus in Lsm1_KD or MajSat RNA OE embryos. Recent studies have determined the vital role of Ezh2 in zygotic H3K27me3 establishment^42,53^. Considering the dramatic increase in EZH2 after PN3 in previous data^42,54^, an insufficient protein level of Ezh2 in early zygotes might serve as the limiting factor for unchanged H3K27me3 under Lsm1 KD or MajSat RNA OE.

The complex relationship between MajSat RNA and embryo development has been studied, with a focus on the stages after the late zygote stage. It is well accepted that MajSat RNA is expressed throughout preimplantation development, peaking at the 2-cell stage, with preferential localization in the male pronucleus in zygotes^43,55^. The Almouzni group reported that MajSat RNA undergoes a strand-specific burst at the 2-cell stage and that interference with its reverse transcripts rather than its forward transcripts at the zygote stage results in obvious developmental arrest^56,57^. In addition, the Torres group found that preimplantation development was compromised by inefficient production of major satellite double-stranded RNA, which is essential for silencing pericentromeric chromatin through the RNAi pathway^16^.

As major components of heterochromatin, the transcripts of pericentromeric chromatin participate in the establishment of heterochromatin, which in turn suppresses the transcription of pericentromeric chromatin^58^. MajSat RNA can interact with SUV39H2 and induce locus-specific H3K9me3 modification by SUV39H2 and HP1 recruitment^59^. However, another study has confirmed the binding property but reported that the enzyme activity of SUV39H2 is attenuated with increasing concentrations of MajSat RNA and that zygote injection of major satellite dsRNA attenuates H3K9me3 levels in the male pronucleus^13^. In this study, we conducted forward-strand RNA injection at the oocyte stage and found H3K9me3 accumulation at the male pronucleus and impaired embryo development. The different readouts of these experiments could be explained in two ways. The different strands of double-stranded MajSat RNA might have different functions. Another key possibility is an effect of the time window. Unlike oocyte injection, zygote injection of MajSat RNA had no vital effect in our study, as at the zygote stage, H3.3 had already been incorporated into the male pronucleus.

Thus, the sharp decrease in MajSat RNA (probably together with other repeat-derived RNAs) upon fertilization seems critical to epigenetic reprogramming and totipotency establishment, and the triggers might be germ cell factors such as Lsm1. As we showed in this study, interference of Lsm1 resulted in impairment of the pronuclear histone profile and subsequent embryo development. LSM1 belongs to the LSM protein family functioning in RNA decay and splicing^37^. LSM1 usually cooperates with LSM2–7 to form a ring-like complex, bind with RNA and further recruit PAT1 to initiate RNA decay^60^. Based on previous studies, LSM1 mainly participates in the decay of mRNA, especially histone mRNA^61^. However, our results provide evidence that repeat element-derived noncoding RNAs are the main targets of LSM1 in zygotes. When Lsm1 is interfered, insufficient major satellite RNA decay leads to excessive H3.1 recruitment and impaired male pronuclear H3.3 incorporation. LSM1-induced MajSat RNA decay is not only indispensable for normal embryo development after fertilization but also insufficient in cloned embryos and could ameliorate developmental defects to some extent. Furthermore, these results strongly suggest that the neglected mRNAs carried by sperm are worthy of additional research.

In summary, our study illustrates the vital role of LSM1-induced MajSat RNA decay in nonequilibrium pronuclear histones and embryo development. Thus, these data provide some insights into epigenetic reprogramming and totipotency establishment upon fertilization. Our results highlight the key roles of repeat element-derived noncoding RNAs in modulating pronucleus-specific epigenetic modifications in early embryos. We believe that exploration of these noncoding RNAs will shed light on the mechanisms of accurate epigenetic control of cell fate determination in the future.

## Methods

### Mice

Specific pathogen-free (SPF)-grade mice, including C57BL/6J, DBA2 and BDF1 mice, were housed in the animal facility at Tongji University, Shanghai, China. Female BDF1 hybrid mice (6–8 weeks old), from which GV- and MII-phase oocytes were collected for the experiment, were obtained by mating female C57BL/6 J mice with male DBA/2 mice. All the mice had free access to food and water, and were housed in 12 hours light/12 hours dark cycle 22.1–22.3 °C and 33–44% humidity. All experiments were performed in accordance with the University of Health Guide for the Care and Use of Laboratory Animals and were approved by the Biological Research Ethics Committee of Tongji University.

### Cell culture

Human HEK293T cell line was purchased from ATCC (American Type Culture Collection) with catalog number CRL-3216. Mouse embryonic fibroblasts (MEFs) were derived from embryos at embryonic day 12.5-13.5 (E12.5-E13.5) in our laboratory. HEK293T cell and MEFs were grown in DMEM (Gibco) supplemented with 10% FBS (Gibco) and 1 mM l-glutamine (Merk Millipore) at 37 °C in a humidified 5% CO_2_ incubator. The cells were cultured in healthy condition without mycoplasma contamination.

### Oocyte and embryo collection

For GV oocyte collection, female BDF1 mice (6–8 weeks old) were superovulated by injection with 6 IU each of pregnant mare serum gonadotropin (PMSG), and at 48 hours after injection, the mice were sacrificed to recover ovaries. The large follicles (larger than 300 μm in diameter) on the ovaries were ruptured in M2 medium containing 2 μM milrinone to release COCs. COCs with more than three layers of unexpanded cumulus cells and containing oocytes >70 μm in diameter and with homogenous cytoplasm were collected.

For MII oocyte collection, female BDF1 mice (6–8 weeks old) were superovulated by injection with 6 IU of pregnant mare serum gonadotropin (PMSG) followed by injection of 6 IU of human chorionic gonadotropin (hCG) (San-Sheng Pharmaceutical) 48 hours later. The superovulated mice were killed 13.5 hours after hCG injection, and the oviductal ampullae were broken in M2 medium to release newly ovulated oocytes.

For zygote collection, superovulated female mice were mated with male BDF1 mice. Then, the zygotes were collected from the oviducts of the female mice at 20 hours after hCG injection.

### GV oocyte injection experiment

For GV oocyte microinjection, fully-grown COCs were pipetted with a small-bore pipette to remove cumulus cells and obtain denuded oocytes, and the oocytes were cultured in maturation medium with 2 μM milrinone (Merck Millipore) to inhibit spontaneous GV breakdown. All injections were performed with a Piezo-driven micromanipulator. Denuded oocytes were injected with 5 to 10 pL of sample per oocyte. After injection, the oocytes were washed and cultured in maturation medium plus 2 μM milrinone at 37 °C with 5% CO_2_ for 2 hours and then transferred to maturation medium.

The microinjected denuded GV oocytes were cultured in maturation medium at 37 °C in a humidified atmosphere of 5% CO_2_ in air. The maturation medium used was tissue culture medium-199 (TCM-199; Gibco) supplemented with 10% (v/v) fetal bovine serum (Gibco), 1 µg/mL 17β-estradiol, 24.2 mg/mL sodium pyruvate, 0.05 IU/mL follicle-stimulating hormone, 0.05 IU/mL luteinizing hormone, and 10 ng/mL epidermal growth factor. MII oocytes were selected 22–24 hours after culture and prepared for in vitro fertilization. The maturation rate was calculated by the number of MII oocyte compared to total injected oocytes.

Then IVF was conducted to matured MII oocytes, sperm collected from the cauda epididymides of adult male BDF1 mice were incubated in G-IVF™ PLUS medium (Vitrolife, 10136) for 20–30 minutes at 37 °C under 5% CO_2_ to induce sperm swim-up. The zona pellucida of each MII oocyte was punched to make a small hole using a Piezo-drill micromanipulator. Oocytes were then placed in the prepared sperm suspension in G-IVF™ PLUS medium (Vitrolife, 10128). At 5 hours post fertilization (hpf), these embryos were washed and cultured in G-IVF™ PLUS medium. The fertilized oocytes were thoroughly washed and cultured in G-1™ PLUS medium at 37 °C under 5% CO_2_.

The blastocyst rate was calculated by the number of blastocysts compared to total fertilized oocytes.

The sequence of the injected oligonucleotides were listed in Supplementary Data 1.

### MII oocyte injection experiment

For MII oocytes zygote microinjection, the oocytes were treated with hyaluronidase (Sigma), and then they were injected with 5 to 10 pL of sample per oocyte. After injection, the oocytes were washed and cultured in CZB medium.

Then IVF was conduct to injected MII oocytes the same as above, and the blastocyst rate was calculated by the number of blastocysts compared to total fertilized oocytes.

The sequence of the injected oligonucleotides were listed in Supplementary Data 1.

### Zygote injection experiment

For zygote microinjection, the zygotes were treated with hyaluronidase (Sigma), and then they were injected with 5 to 10 pL of sample per zygote. After injection, the zygotes were washed and cultured in G-1™ PLUS medium at 37 °C under 5% CO_2_. The blastocyst rate was calculated by the number of blastocysts compared to total fertilized oocytes.

The sequence of the injected oligonucleotides were listed in Supplementary Data 1.

### In vitro transcription and preparation of RNAs for microinjections

The sequences of 3XFlag-tagged Lsm1 and 471 bp MajSat RNA driven by the T7 promoter were synthesized and ligated into pUC57_Plasmid purchased from Genewiz. The sequence of MajSat RNA is 471 bp GAST sequence which can be found at Repbase (https://www.girinst.org/repbase/update/index.html). The plasmids were linearized and subjected to phenol–chloroform extraction and ethanol precipitation as templates for in vitro transcription. Mouse 3XFlag-tagged Lsm1 mRNA was synthesized with an mMESSAGE mMACHINE™ T7 ULTRA Transcription Kit (Thermo Fisher Scientific, AM1345) according to the manufacturer’s instructions. Mouse 471 bp MajSat RNA was synthesized with a MEGAscript™ T7 Transcription Kit (Thermo Fisher Scientific, AM1333) according to the manufacturer’s instructions. Both RNAs were recovered by lithium chloride precipitation and resuspended in nuclease-free water. The integrity of the synthesized mRNA was confirmed by electrophoresis. The precipitated RNA was dissolved in H_2_O at several concentrations and stored at −80 °C until use.

To prepare RNA for microinjection, 3XFlag-tagged Lsm1 mRNA was diluted to 100 ng/μL, and MajSat RNA was diluted to 150 ng/μL before injection.

### Immunofluorescent staining and confocal microscopy for oocytes and embryos

Oocytes and embryos were fixed with 4% paraformaldehyde (Sigma) overnight at 4 °C and then permeabilized with 0.5% Triton X-100 for 5 minutes at room temperature. The samples were blocked with 3% bovine serum albumin (BSA) (Sigma) at 25 °C for 1–2 hours and then incubated with the primary antibodies anti-H3K9me3 (Active Motif, 39161, 1:100), anti-H3K27me3 (Diagenode, C15410195, 1:200), anti- H3K14ac (Active Motif, 39599, 1:50), anti- CBX1 (Proteintech, 10241-2-AP, 1:50), anti- H3.1/3.2 (Cosmo Bio, CAC-CE-039B, 1:100), anti- H3.3 (Thermo Fisher Scientific, MA5-24667, 1:100), anti- ATRX (Santa Cruz, sc-55584, 1:100) overnight at 4 °C. After washing three times with TBST, the samples were incubated with the appropriate secondary antibodies -Alexa Fluor® 594 Donkey Anti-Rabbit IgG (H + L) Antibody (Fisher Scientific, A-21207, 1:500) and Alexa Fluor® 594 Donkey Anti-Mouse IgG (H + L) Antibody (Fisher Scientific, A-21203, 1:500) - for 1–2 hours. The nuclei were stained with 4′,6-diamidino-2-phenylindole (DAPI) for 5 minutes. The oocytes and embryos were mounted on glass slides with SlowFade Gold Antifade Reagent (Life Technologies) and examined under a confocal laser scanning microscope (Zeiss LSM880, Carl Zeiss).

Fluorescence intensity quantitative analysis was conduct according to previous method^62–64^. Brifely, for each experimental series, all the images were acquired with identical settings. A single plane with maximum amount of chromatin and maximum fluorescence intensity was selected to take photograph for further analysis. Relative fluorescence intensities were measured on the raw images of male and female pronucleus regions by cropping the exact areas using the ZEN software (Carl Zeiss Microscopy) under fixed parameters across all the samples.

### Sample harvest for total RNA-seq, RIP-seq

The zona pellucidae of the zygotes was removed with 0.5% pronase E (Sigma) and then incubated in Ca^2+^-free CZB medium for 10−20 minutes. Polar bodies were removed by gentle pipetting using a fire-polished glass needle. After 3 washes with PBS with 0.5% BSA, each sample was frozen in liquid nitrogen and stored at −80 °C for later manipulation.

### RNA-Seq for small amounts of RNA

We followed the methods described previously for single-cell RNA-seq^65^. Briefly, 2–5 embryos were collected as one replicate. The zona pellucidae of the embryos were removed with 0.5% protease E. The embryos were washed 5–6 times in 0.5% BSA in PBS before being placed into cell lysis buffer. After incubation at 70 °C for 90 seconds, reverse transcription for first-strand cDNA synthesis was performed using SuperScript III Reverse Transcriptase (Thermo Fisher Scientific, 18080044). After second-strand cDNA synthesis, the cDNA library was amplified for 16–18 cycles based on the embryo number. For library construction, the amplified cDNA was sheared into 200–300 bp fragments using the Covaris S220 system. The libraries were generated using a KAPA HyperPlus Library Preparation Kit (KAPA Biosystems, KK8504) following the manufacturer’s instructions. The libraries were sequenced on an Illumina NovaSeq 6000 platform with paired ends and 150-bp read lengths at Berry Genomics.

### RIP-seq

RIP was performed as described previously with some modifications^66^. Briefly, 300 oocytes were lysed using PLB (15 mM Tris-HCl pH 7.5, 50 mM NaCl, 5 mM MgCl_2_, 1% Triton, 0.15 mM Na_3_VO_4_, 10 mM β-glycerophosphate, 1 mM MDTT (Sigma–Aldrich, 646563), protease inhibitors (Sigma–Aldrich, P8340), 40 U RNaseOUT (Invitrogen, 10777–019), 100 μg/mL cycloheximide (Sigma–Aldrich, C7698) and 2 mM vanadyl ribonucleoside (NEB, S1402S). Ten percent of the lysate was collected as input, and the rest was incubated with ANTI-FLAG® M2 Affinity Gel (Millipore, A4596) at 4 °C on a rotor overnight. The gels were washed with PLB for four times each time 10 minutes. In the final wash, 1 M urea was added. RNAs from the input and IP samples were isolated using TRIzol Reagent and chloroform, except that a 1/10 volume of 3 M NaAc and 1 μL of glycogen were added to the aqueous phase of each sample. The RNAs were then precipitated with isopropanol and washed twice with 75% ethanol before they were eluted with nuclease-free water. Finally, the purified RNAs were subjected to library generation using a SMARTer Stranded Total RNA-Seq Kit (Takara Bio, 634412). The libraries were sequenced on an Illumina NovaSeq 6000 platform with paired ends and 150-bp read lengths at Berry Genomics.

### RNA isolation and real-time RT–PCR

Total RNA was extracted with TRIzol reagent according to the manufacturer’s instructions. cDNA was prepared with a SuperScript III First-Strand Synthesis System using random hexamer oligonucleotide primers. qPCR was carried out using TB Green Premix Ex Taq II (Takara Bio, RR820A) and monitored with a 7500 Fast Real-Time PCR System, and three technical replicates were performed for each sample. Data were analyzed using DART-PCR^67^. The relative expression level of each gene was normalized to the level of the reference gene Hprt or β-Actin. Primers were listed in Supplementary Data 1.

### Plasmid transfection and MS2bp-YFP RNA pulldown

An MS2bp-MS2bs-based RIP assay was performed as described previously^68^. In brief, pcDNA3.1-MajSAT-MS2bs, pcDNA3.1-H3.1/H3.3 and MS2bp-YFP plasmids were constructed. Transient plasmid transfection of HEK293T cells was performed with Lipofectamine 2000 (Invitrogen). 48 hours after transfection, cells were harvested for crosslinking with 600 mJ/cm^2^ of 254 nm UV light and then lysed by lysis buffer (50 mM Tris-HCl [pH 7.5], 150 mM NaCl, 10% glycerol, and 0.5% NP-40, with protease and RNase inhibitors added before use). After centrifugation at 12,000 × g for 10 minutes, the supernatant was subjected to preclearing by incubation with Dynabeads™ Protein A (Thermo Fisher Scientific, 10002D) with rotation at 4 °C for 30 minutes. Ten percent of the supernatant was kept as input, and the rest was aliquoted equally into two parts for GFP antibody which can specifically bind with GFP (Invitrogen, A11122) and IgG (Sigma-Aldrich, 12-370) antibody incubation. After 2 hours of rotation at 4 °C, protein A beads were added, and the samples were incubated with rotation at 4 °C overnight. The beads were washed with wash buffer (60 mM Tris–HCl [pH 7.4], 300 mM KCl, 12 mM MgCl_2_, 1% NP40, 1 mM DTT, RNase inhibitors, and 100 μg/mL cycloheximide) for three times each time 10 minutes. In the final two washes, 1 M urea was added. SDS sample buffer was added to the beads, and the eluates were used for western blot analysis.

### Western blotting

The samples were lysed with SDS sample buffer and heated for 5 minutes at 95 °C. The proteins were separated by 10% SDS–PAGE and electrophoretically transferred to PVDF membranes, which were blocked in TBST containing 5% BSA for 1 hours. The membranes were then incubated with primary antibodies at 4 °C overnight. The primary antibodies were as follows: anti-H3.1 (Novus Biologicals, NBP2-75524, 1:1000), anti-H3.3 (Thermo Fisher Scientific, MA5-24667, 1:1000), anti-TUBULIN (Proteintech, 66031-1-Ig, 1:2000), anti-SNRP70 (Abcam, ab83306, 1:1000) and anti-GAPDH (Proteintech, 10494-1-AP, 1:1000). The membranes were washed in TBST, incubated with an HRP-linked secondary antibody - Amersham ECL Rabbit IgG, HRP-linked whole Ab (Cytiva, NA934, 1:10,000) and Amersham ECL Mouse IgG, HRP-linked whole Ab (Cytiva, NA931, 1:10,000) - for 1 hour at room temperature, and washed three times with TBST. The protein signals were measured using High-Sig ECL Western Blot Substrate (Tanon) and visualized with a ChemiDoc MP imaging system. The uncropped and unprocessed scans of the blots were provided in the Source Data file.

### MajSat RNA decay assay

An RNA decay assay was performed as previously described with some modifications^69^. In brief, zygotes were treated with actinomycin D (Sigma, A9415), and then samples were collected at 0 hour, 2 hours, 4 hours, and 8 hours after actinomycin D treatment. Total RNA was extracted and purified using the same method as that used for RIP-seq, and cDNA was prepared with a SuperScript III First-Strand Synthesis System using random hexamer oligonucleotide primers. MajSat RNA was detected by real-time PCR analysis, and the expression was normalized to the expression of the control gene 18S.

### Nuclear and cytoplasmic extraction

Nuclear and cytoplasm of MEF cells were separated and extracted by NE-PER™ Nuclear and Cytoplasmic Extraction Reagents (Thermo Scientific, 78833) according to the manufacturer’s protocol. The protein level of SNRP70 (cytoplasmic) and GAPDH (nuclear) in cytoplasmic and nuclear fraction was checked to estimate separation efficiency.

### RNA-Seq and RIP-seq data analysis

All sequencing data from the Illumina HiSeq X-ten platform or Illumina NovaSeq 6000 (Berry Genomics) were trimmed with Cutadapt (v2.3) to remove adapter sequences and low-quality reads. The quality control of RNA-Seq and RIP-seq data was performed using FastQC (v0.11.8) and MultiQC (v1.11) and all trimmed data passed this step. The trimmed sequencing reads were further mapped to the mm10 reference genome using STAR (v 2.7.8a) with the “–outFilterIntronMotifs RemoveNoncanonicalUnannotated” parameters. The bigWig file for each sample was normalized according to the sequencing depth using the BEDtools (v2.29.2) genomecov function, for visualization in IGV (v2.11.0). The expression level of each transcript was quantified as transcript per million (TPM) using Salmon (v0.8.2), including RefSeq annotated RNA transcripts and transposon sequences downloaded from Repbase (v25.06). We used the pipeline, PipeRNAseq (https://github.com/sunyumail93/PipeRNAseq), in our data processing to enhance the automation and reproducibility of the above steps. Salmon output files were further imported in R 3.5.0 using the tximport (v1.20.0) package for downstream analysis. Differential expression analysis was performed using DESeq2 (v1.32.0) package with adjusted *p* value < 0.05. Functional enrichment analysis was performed using clusterProfiler (v4.0.0) package, and visualized using “enrichGO”, “barplot” and “dotplot” functions. We focused on Gene Ontology terms including Molecular Function, Cellular Component, and Biological Process. We also summarized the downstream analysis into a wrapper R package, easyRNAseqDE (https://github.com/sunyumail93/easyRNAseqDE). To specifically visualize RNA-Seq and RIP-seq data mapped to each transposon family, paired-ends reads were mapped directly to transposon consensus sequences using bowtie2 (v2.3.5.1). The un-mapped reads were filtered out using Samtools (v1.11), and mapped fragments were inferred from the mapped read pairs. We finally plotted the mapped fragments on each transposon consensus sequence and visualized the signals using R (v3.5.0).

### Statistical analysis

All statistical analyses were performed with GraphPad Prism 7.0 software, except genome-wide data analyses, which were performed in R/Bioconductor. Details of the individual tests were outlined within each figure legend, including the number and type of replicates (*n*), and the error is reported as either the standard deviation (SD) or standard error of the mean (SEM). For all statistics, significance is indicated as follows: *p < 0.05, **p < 0.01 and ***p < 0.001, ns means not significant. Significance was calculated by two-sided Student’s *t* test unless specifically noted otherwise in the legend. Welch’s correction was applied to *t* tests whenever the variance was unequal between conditions.

### Reporting summary

Further information on research design is available in the Nature Portfolio Reporting Summary linked to this article.

## Supplementary information

Supplementary Information

Description of Additional Supplementary Files

Supplementary Data 1

Reporting Summary

## Data Availability

The total RNA-seq and RIP-seq data generated in this study have been deposited in the Gene Expression Omnibus (GEO) database under accession code GSE179556. All other data supporting the findings of this study are available from the corresponding authors on reasonable request. Source data for the figures and supplementary figures are provided as a Source Data file. Source data are provided with this paper.

## Source data

Source Data
