# Supplementary material for: LSM1-mediated Major Satellite RNA decay is required for nonequilibrium histone H3.3 incorporation into parental pronuclei
Source: Nat Commun. 2023 Feb 21;14:957. doi: 10.1038/s41467-023-36584-z (PMC9944933; doi:10.1038/s41467-023-36584-z)
Supplement: Supplementary file 1 — Supplementary Information [file 41467_2023_36584_MOESM1_ESM.pdf]

Supplementary Information

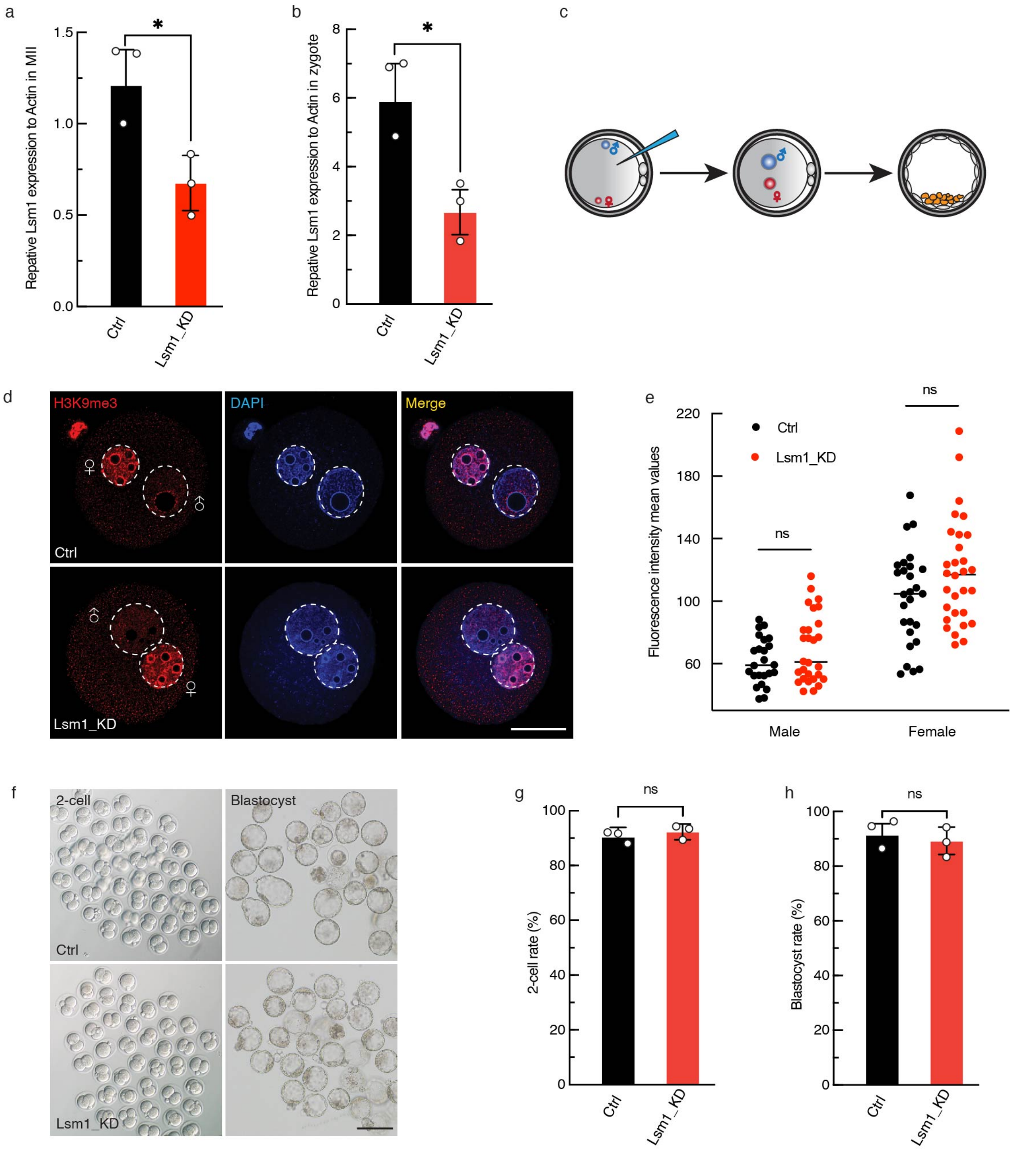

Supplementary Fig. 1 | LSM1 is essential for embryo development, Related to Fig. 1
a, qPCR validation of Lsm1 knockdown efficiency in MII oocytes. The statistical data are expressed as mean ± SEM, *p < 0.05 (p = 0.0471) by two-sided Student's t test for each comparison. Each reaction for qPCR analysis contains 3 biological replicates, with each replicate from 50 oocytes.
b, qPCR validation of Lsm1 knockdown efficiency in zygotes. The statistical data are expressed as mean ± SEM, *p < 0.05 (p = 0.0429) by two-sided Student's t test for each

comparison. Each reaction for qPCR analysis contains 3 biological replicates, with each replicate from 50 zygotes.

c, Scheme for Lsm1 zygote-stage knockdown experiment. Lsm1 siRNA was injected at early zygote stage, then the late zygotes were used for H3K9me3 immunostaining and the further development analysis.

d-e, Immunostaining of H3K9me3 in zygote-stage knockdown experiment. Images of H3K9me3 (d) and fluorescence intensity mean values (e) in pronucleus of Ctrl and Lsm1_KD zygotes were represented. Scale bars, 20 µm. Representative images from 3 biological replicates, with each replicate originating from at least 10 zygotes. The statistical data are expressed as mean ± SEM, ns (male p = 0.9248, female p = 0.9522) means not significant by two-sided Student's t test for each comparison. Each treatment contains 3 biological replicates, with each replicate originating from approximately 30 zygotes.

f-h, Quantification of embryo development competence in response to Lsm1 knockdown. Images of 2-cell embryos and blastocysts (f), statistics for 2-cell rate (g) and blastocyst rate (h) for Ctrl and Lsm1_KD group were represented. Scale bars, 100 µm. Representative images from 3 biological replicates, with each replicate originating from approximately 35 injected zygotes (g). The statistical data are expressed as mean ± SEM, ns (p = 0.7077 for g, p = 0.7508 for h) means not significant by two-sided Student's t test for each comparison. Each treatment contains 3 biological replicates, with each replicate originating from approximately 35 injected zygotes.

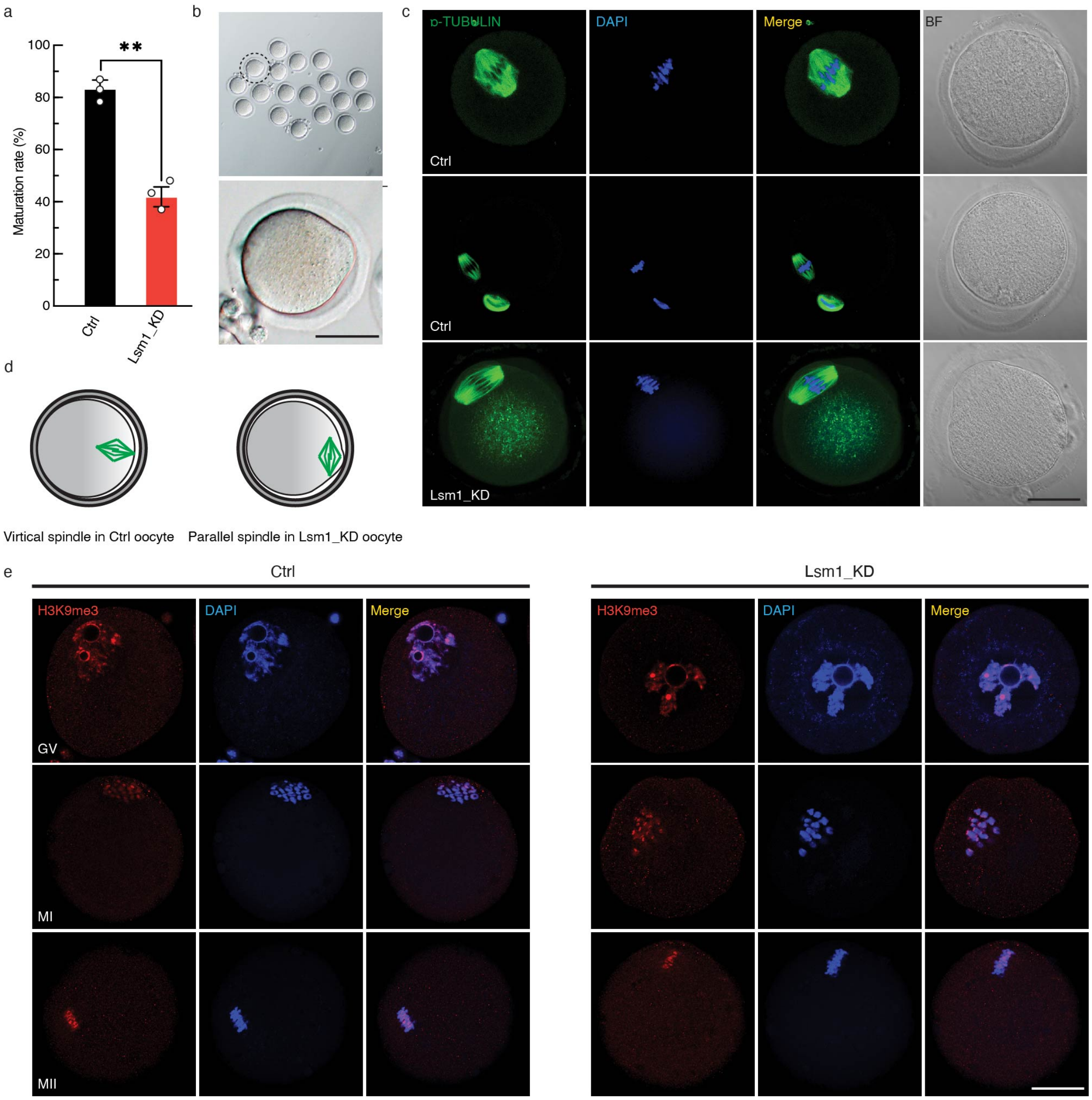

Supplementary Fig. 2 | LSM1 is essential for oocyte maturation, Related to Fig. 1

a, Statistics for oocyte maturation rate in response to Lsm1 KD. The statistical data are expressed as mean ± SEM, $^{**}p < 0.01$ ($p = 0.0013$) by two-sided Student's t test for each comparison. Each treatment contains 3 biological replicates, with each replicate originating from approximately 35 injected GV-oocytes.

b, Representative images of immature oocytes in Lsm1_KD group. Scale bars, 50 µm. Representative images from 3 biological replicates, with each replicate originating from approximately 35 injected GV-oocytes.

c, Immunostaining for spindle localization in Ctrl and Lsm1_KD oocytes. Scale bars, 20 µm. Representative images from 3 biological replicates, with each replicate containing approximately 10 oocytes.

d, Model for spindle localization in Ctrl and Lsm1_KD oocytes.

e, Immunostaining for H3K9me3 in Ctrl and Lsm1_KD oocytes at GV, MI and MII stage. Scale bars, 20 µm. Representative images from 3 biological replicates, with each replicate containing approximately 10 oocytes.

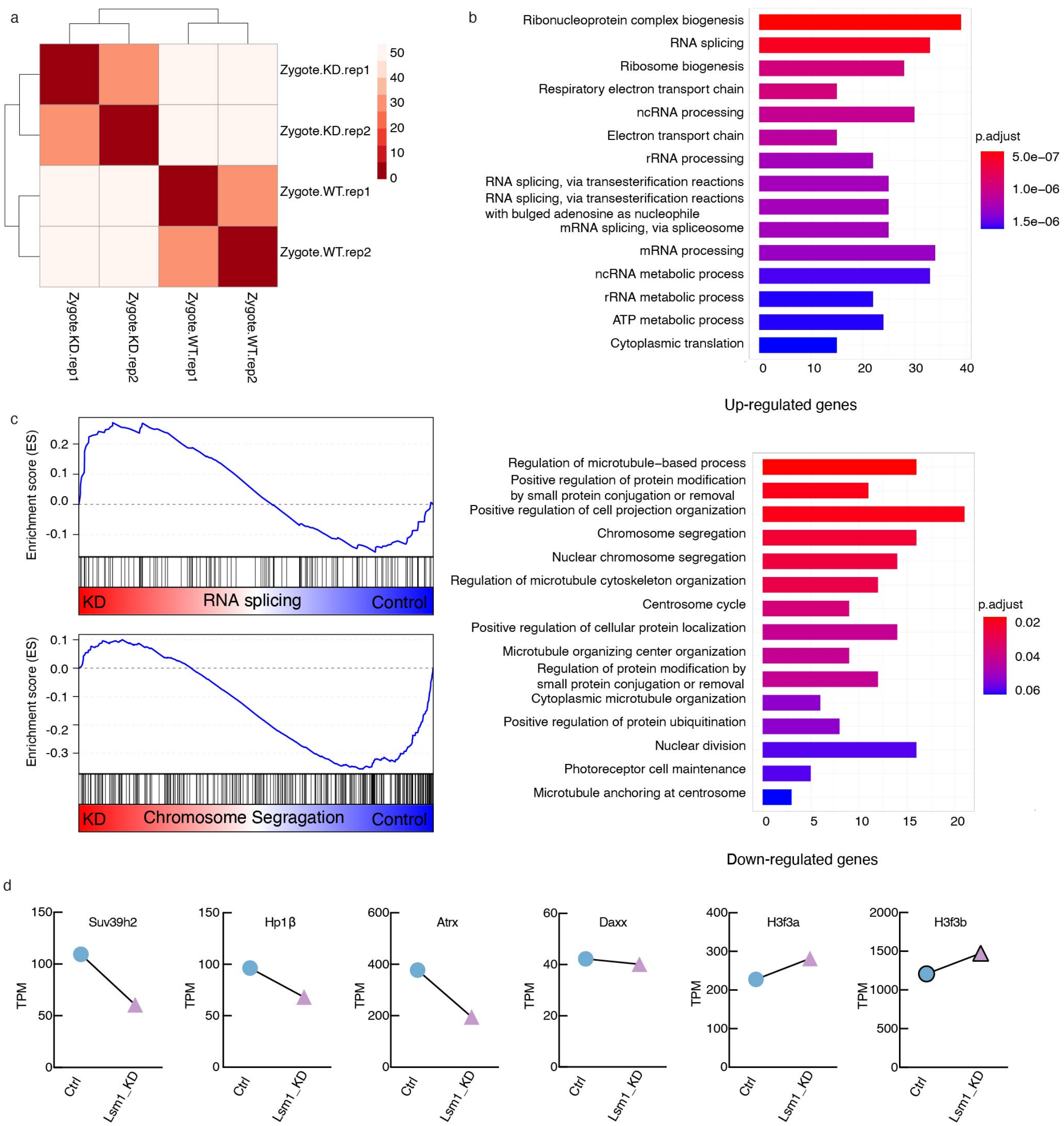

Supplementary Fig. 3 | Total RNA-seq analysis for Lsm1_KD zygotes, Related to Fig. 2

a, Unsupervised hierarchical clustering of samples using Pearson Correlation coefficients showing Lsm1_KD zygotes displayed a distinct gene expression profile compared with the control ones. n = 2 biologically independent samples.

b, Gene Ontology analysis of molecular function for up-regulated and down-regulated genes in Lsm1_KD zygotes. One-sided hypergeometric test was used to determine the adjusted *p*-value using Benjamini-Hochberg correction, n = 2 biologically independent samples.

c, GSEA plot showing up-regulation of RNA splicing pathway and down-regulation of chromosome segregation processing pathway under Lsm1 KD.

d, The TPM values of H3K9me3 modification related genes and H3 genes in Lsm1_KD zygotes compared with control.

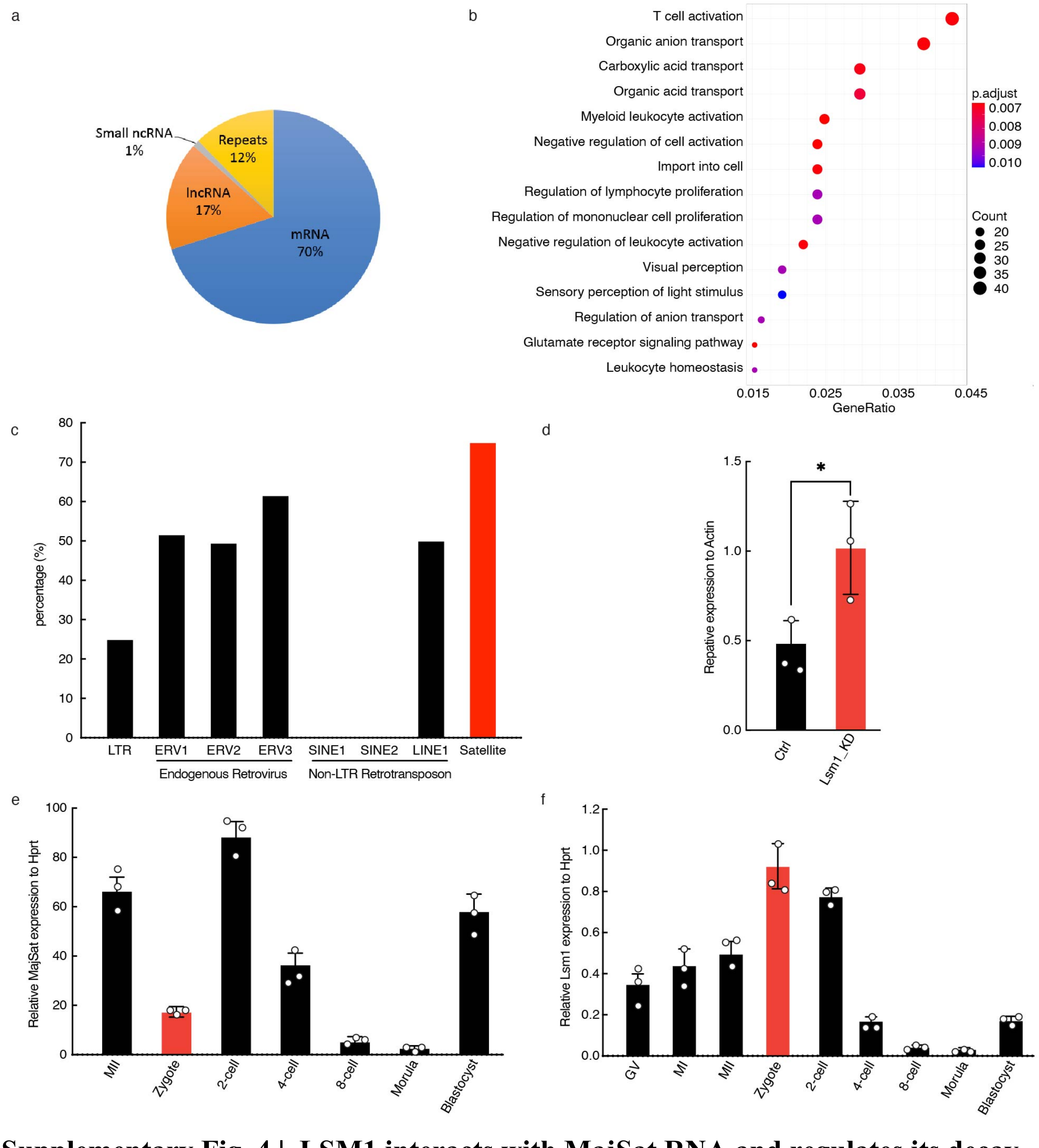

Supplementary Fig. 4 | LSM1 interacts with MajSat RNA and regulates its decay, Related to Fig. 3

a, Pie chart showing the composition of LSM1 binding RNAs in zygote.

b, Gene Ontology analysis of biological process for LSM1 binding genes in zygote. One-sided hypergeometric test was used to determine the adjusted *p*-value using Benjamini-Hochberg correction, n = 2 biologically independent samples.

c, Bar plot showing the percentage of LSM1 binding repeats derived RNAs by item number in different repeats families.

d, qPCR examination of MajSat RNA in Lsm1_KD zygotes. The statistical data are expressed as mean ± SEM, *$p < 0.05$ ($p = 0.4873$) by two-sided Student's *t* test for each

comparison. Each reaction for qPCR analysis contains 3 biological replicates, with each replicate from 50 zygotes.

e, qPCR examination of MajSat RNA during oocyte maturation and early embryo development. Each reaction for qPCR analysis contains 3 biological replicates, with each replicate from 50 oocytes/embryos.

f, qPCR examination of Lsm1 mRNA during oocyte maturation and early embryo development. Each reaction for qPCR analysis contains 3 biological replicates, with each replicate from 50 oocytes/embryos.

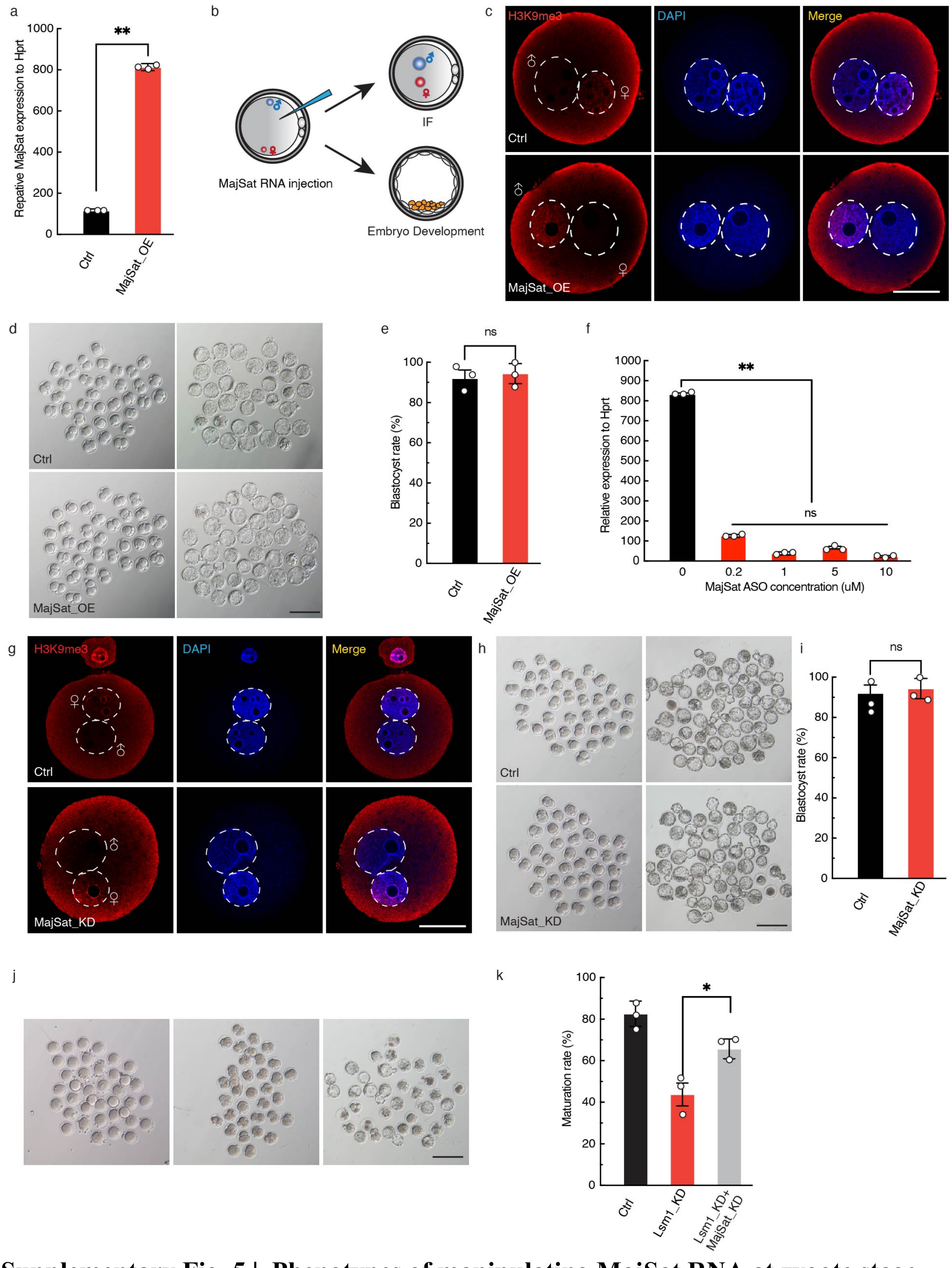

Supplementary Fig. 5 | Phenotypes of manipulating MajSat RNA at zygote stage, Related to Fig. 4

a, qPCR validation of major satellite RNA overexpression efficiency. The statistical data are expressed as mean ± SEM, $^{**}p < 0.01$ ($p = 0.0011$) by two-sided Student's t test for each comparison. Each reaction for qPCR analysis contains 3 biological replicates, with each replicate from 50 zygotes.

b, Scheme for MajSat RNA zygote-stage overexpression experiments. MajSat in vitro transcribed RNA was injected at early zygote stage, then the late zygotes were used for H3K9me3 immunostaining and the further development analysis.
c, Immunostaining for H3K9me3 in Ctrl and MajSat_OE zygote. Scale bars, 20 μm. Representative images from 3 biological replicates, with each replicate containing approximately 30 zygotes.
d-e, Quantification of embryo development competence in response to MajSat overexpression at zygote stage. Images of 2-cell embryos and blastocysts for the Ctrl and MajSat_OE groups (d) and statistics for blastocyst rates (e) were represented. Scale bars, 100 μm. Representative images from 3 biological replicates, with each replicate originating from approximately 35 injected zygotes (d). The statistical data are expressed as mean ± SEM, ns ($p = 0.8120$) means not significant by two-sided Student's *t* test for each comparison. Each treatment contains 3 biological replicates, with each replicate originating from approximately 35 injected zygotes.
f, qPCR validation of MajSat RNA knockdown efficiency. The statistical data are expressed as mean ± SEM, $^{**}p < 0.01$ ($p = 0.0096$) and ns ($p = 0.6100$) means not significant by one-way ANOVA test. Each reaction for qPCR analysis contains 3 biological replicates, with each replicate from 50 zygotes.
g, Immunostaining for H3K9me3 in Ctrl and MajSat_KD zygote. Scale bars, 20 μm. Representative images from 3 biological replicates, with each replicate containing approximately 30 zygotes.
h-I, Quantification of embryo development competence in response to MajSat overexpression at zygote stage. Images of 2-cell embryos and blastocysts for the Ctrl and MajSat_KD groups (h) and statistics for blastocyst rates (i) were represented. Scale bars, 100 μm. Representative images from 3 biological replicates, with each replicate originating from approximately 35 injected zygotes (h). The statistical data are expressed as mean ± SEM, ns ($p = 0.7400$) means not significant by two-sided Student's *t* test for each comparison. Each treatment contains 3 biological replicates, with each replicate originating from approximately 35 injected zygotes.
j, Representative images of oocytes, 2-cell embryos and blastocysts for Lsm1_KD + Majsat_KD group, scale bars, 100 μm. Representative images from 3 biological replicates, with each replicate originating from approximately 35 injected GV-oocytes.
k, Statistics for maturation rate in Ctrl, Lsm1_KD and Lsm1_KD + MajSat_KD zygotes. The statistical data are expressed as mean ± SEM, $^{*}p < 0.05$ ($p = 0.0337$) by one-way ANOVA test. Each treatment contains 3 biological replicates, with each replicate originating from approximately 35 injected GV-oocytes.

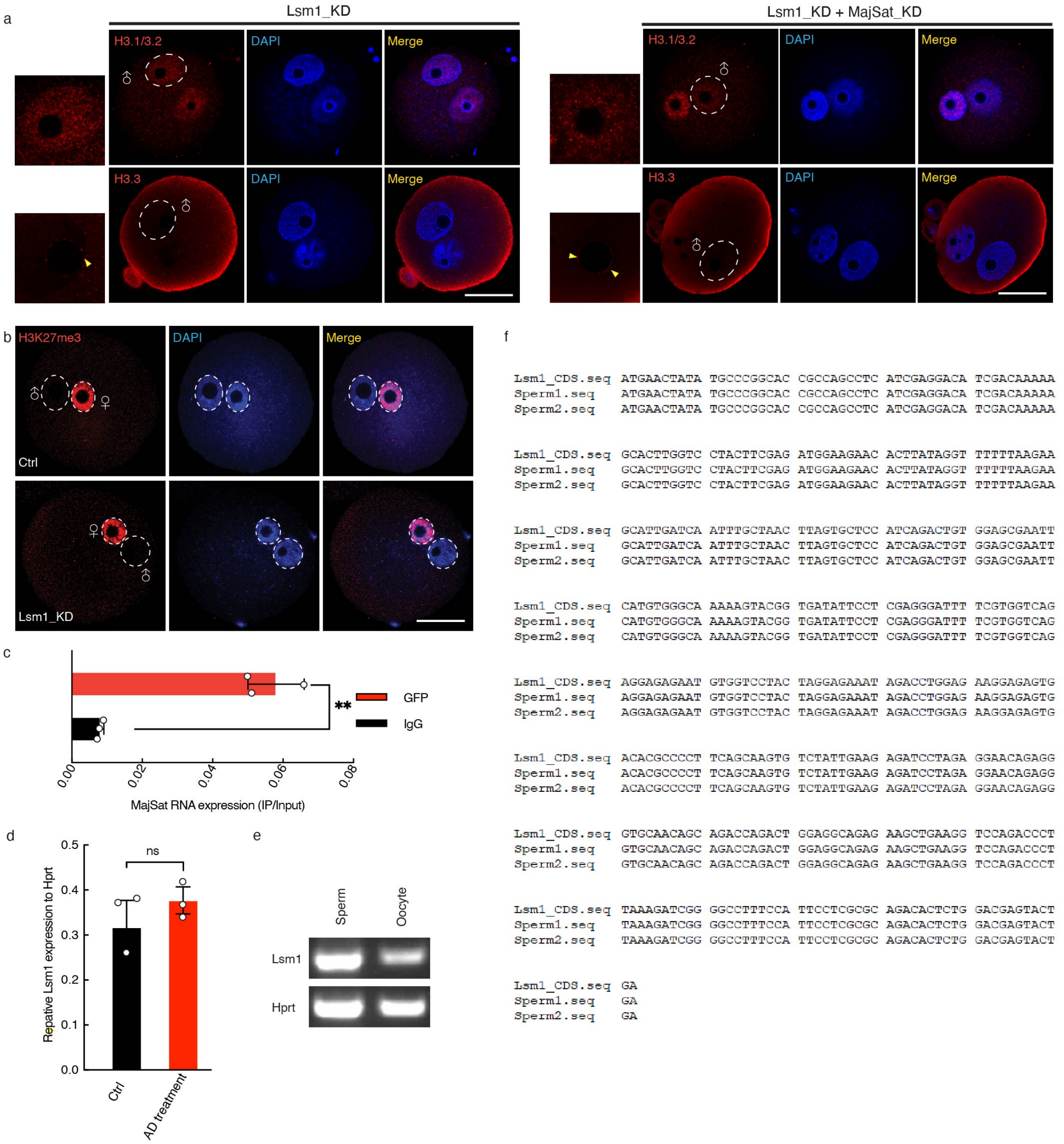

Supplementary Fig. 6 | MajSat RNA regulates H3.1/3.2 incorporation and Lsm1 exists in both gametes, Related to Fig. 5 and Fig. 6

a, Immunostaining for H3.1/3.2 and H3.3 in Lsm1_KD and Lsm1_KD + MajSat_KD zygotes. Scale bars, 20 µm. Representative images from 3 biological replicates, with each replicate containing approximately 30 zygotes.

b, Immunostaining for H3K27me3 in Ctrl and Lsm1_KD zygotes. Scale bars, 20 µm. Representative images from 3 biological replicates, with each replicate containing approximately 30 zygotes.

c, qPCR validation of pulldown efficiency. The statistical data are expressed as mean ± SEM, $^{**}p < 0.01$ ($p = 0.0034$) by two-sided Student's t test for each comparison. Each reaction for qPCR analysis contains 3 biological replicates, with each replicate from 5*10^7 cells.

d, qPCR examination of Lsm1 mRNA level in Actinomycin D (AD)-treated zygotes. The statistical data are expressed as mean ± SEM, ns (p = 0.4216) means not significant by two-sided Student's *t* test for each comparison. Each reaction for qPCR analysis contains 3 biological replicates, with each replicate from 50 zygotes.

e, Images of agarose gel showing RT–PCR amplification of full-length Lsm1 mRNA from sperm and oocytes. Representative images from 3 biological replicates.

f, Sequence comparing of the first-generation sequencing data of the sperm-harbored Lsm1 mRNA and the intact Lsm1 cDNA.
