# Supplementary material for: LSM1-mediated Major Satellite RNA decay is required for nonequilibrium histone H3.3 incorporation into parental pronuclei
Source: Nat Commun. 2023 Feb 21;14:957. doi: 10.1038/s41467-023-36584-z (PMC9944933; doi:10.1038/s41467-023-36584-z)
Supplement: Supplementary file 2 — Description of Additional Supplementary Files [file 41467_2023_36584_MOESM2_ESM.docx]

File name: Supplementary Data 1

Description: The sequence information for qRT-PCR, PCR Primers and Oligonucleotides used in this study.
