# Supplementary material for: LSM1-mediated Major Satellite RNA decay is required for nonequilibrium histone H3.3 incorporation into parental pronuclei
Source: Nat Commun. 2023 Feb 21;14:957. doi: 10.1038/s41467-023-36584-z (PMC9944933; doi:10.1038/s41467-023-36584-z)
Supplement: Supplementary file 4 — Reporting Summary [file 41467_2023_36584_MOESM4_ESM.pdf]

nature portfolio

Corresponding author(s): Lan Kang, Shaorong Gao

Last updated by author(s): Nov 8, 2022

Reporting Summary

Nature Portfolio wishes to improve the reproducibility of the work that we publish. This form provides structure for consistency and transparency in reporting. For further information on Nature Portfolio policies, see our Editorial Policies and the Editorial Policy Checklist.

Statistics

For all statistical analyses, confirm that the following items are present in the figure legend, table legend, main text, or Methods section.

n/a	Confirmed	
☐	☒	The exact sample size (*n*) for each experimental group/condition, given as a discrete number and unit of measurement
☐	☒	A statement on whether measurements were taken from distinct samples or whether the same sample was measured repeatedly
☐	☒	The statistical test(s) used AND whether they are one- or two-sided<br>*Only common tests should be described solely by name; describe more complex techniques in the Methods section.*
☒	☐	A description of all covariates tested
☐	☒	A description of any assumptions or corrections, such as tests of normality and adjustment for multiple comparisons
☐	☒	A full description of the statistical parameters including central tendency (e.g. means) or other basic estimates (e.g. regression coefficient) AND variation (e.g. standard deviation) or associated estimates of uncertainty (e.g. confidence intervals)
☐	☒	For null hypothesis testing, the test statistic (e.g. *F*, *t*, *r*) with confidence intervals, effect sizes, degrees of freedom and *P* value noted<br>*Give P values as exact values whenever suitable.*
☒	☐	For Bayesian analysis, information on the choice of priors and Markov chain Monte Carlo settings
☒	☐	For hierarchical and complex designs, identification of the appropriate level for tests and full reporting of outcomes
☐	☒	Estimates of effect sizes (e.g. Cohen's *d*, Pearson's *r*), indicating how they were calculated

Our web collection on statistics for biologists contains articles on many of the points above.

Software and code

Policy information about availability of computer code

Data collection	Microscopy images were taken using Zeiss LSM880 Microsystems. RNA-seq and RIP-seq were performed at Berry Genomics Corporation.
Data analysis	Images were processed with the ZEN software (Zeiss LSM880).<br>Statistical analyses were carried out using the Prism software (GraphPad v9.0).<br>All sequencing data were trimmed with Cutadapt (v2.3) to remove adapter sequences and low-quality reads. The quality control of RNA-Seq and RIP-seq data was performed using FastQC (v0.11.8) and MultiQC (v1.11) and all trimmed data passed this step. The trimmed sequencing reads were further mapped to the mm10 reference genome using STAR (v 2.7.8a) with the " --outFilterIntronMotifs RemoveNoncanonicalUnannotated" parameters. The bigWig file for each sample was normalized according to the sequencing depth using the BEDtools (v2.29.2) genomecov function, for visualization in IGV (v2.11.0). The expression level of each transcript was quantified as transcript per million (TPM) using Salmon (v0.8.2), including RefSeq annotated RNA transcripts and transposon sequences downloaded from Repbase (v25.06). Salmon output files were further imported in R 3.5.0 using the tximport (v1.20.0) package for downstream analysis. Differential expression analysis was performed using DESeq2 (v1.32.0) package with adjusted p value < 0.05. Functional enrichment analysis was performed using clusterProfiler (v4.0.0) package, and visualized using"enrichGO", "barplot" and "dotplot" functions. We focused on Gene Ontology terms including Molecular Function, Cellular Component, and Biological Process. To specifically visualize RNA-Seq and RIP-seq data mapped to each transposon family, paired-ends reads were mapped directly to transposon consensus sequences using bowtie2 (v2.3.5.1). The un-mapped reads were filtered out using Samtools (v1.11), and mapped fragments were inferred from the mapped read pairs. We finally plotted the mapped fragments on each transposon consensus sequence and visualized the signals using R (v3.5.0).

For manuscripts utilizing custom algorithms or software that are central to the research but not yet described in published literature, software must be made available to editors and reviewers. We strongly encourage code deposition in a community repository (e.g. GitHub). See the Nature Portfolio guidelines for submitting code & software for further information.

Data

Policy information about availability of data

All manuscripts must include a data availability statement. This statement should provide the following information, where applicable:

- Accession codes, unique identifiers, or web links for publicly available datasets
- A description of any restrictions on data availability
- For clinical datasets or third party data, please ensure that the statement adheres to our policy

The main data supporting the findings of this study are available within the article and its Supplementary Information files. All the RNA-Seq data and RIP-Seq data generated during this study are available at GEO: GSE179556 GSE179556 [https://www.ncbi.nlm.nih.gov/geo/query/acc.cgi?acc=GSE179556].There is no restrictions on data availability. Any additional data relevant to this manuscript are available from the authors upon reasonable request.

Field-specific reporting

Please select the one below that is the best fit for your research. If you are not sure, read the appropriate sections before making your selection.

☒ Life sciences ☐ Behavioural & social sciences ☐ Ecological, evolutionary & environmental sciences

For a reference copy of the document with all sections, see nature.com/documents/nr-reporting-summary-flat.pdf

Life sciences study design

All studies must disclose on these points even when the disclosure is negative.

Sample size	2-5 zygotes per reaction for RNA-sequencing (Fan et al., Genome Biology, 2015) , 300 zygotes per reaction for RIP-seq (Sousa Martins et al., Journal of Cell Science, 2016) and 50 zygotes per reaction for qPCR analysis (TRIzol™ Reagent, Invitrogen). 5*10^7 cells were collected for MS2bp-YFP RNA pull down experiment (Wang et al., Developmental Cell, 2013). For embryo development and immunofluorescent staining analysis, sample sizes were determined without statistical measures, but based on prior experience with the specific experiments and widely used sizes in relevant publications within this field of research in order to ensure that it will be appropriate for statistical analysis.
Data exclusions	No data were excluded from the analyses.
Replication	For each representative image, experiments were performed at least three times with similar results. For Fluorescence intensity quantitative analysis at least 15 clear photos were used. RNA-seq and RIP-seq were performed 2 times with the number of replications. All attempts at replication were successful.
Randomization	Samples were randomly allocated into experimental groups prior to treatment.
Blinding	The investigators were not blinded during data collection and analysis. Because experiments execution, data collection and result analysis were usually carried out by the same person, therefore no blinding was used.

Reporting for specific materials, systems and methods

We require information from authors about some types of materials, experimental systems and methods used in many studies. Here, indicate whether each material, system or method listed is relevant to your study. If you are not sure if a list item applies to your research, read the appropriate section before selecting a response.

Materials & experimental systems

n/a	Involved in the study	
☐	☒	Antibodies
☐	☒	Eukaryotic cell lines
☒	☐	Palaeontology and archaeology
☐	☒	Animals and other organisms
☒	☐	Human research participants
☒	☐	Clinical data
☒	☐	Dual use research of concern

Methods

n/a	Involved in the study	
☒	☐	ChIP-seq
☒	☐	Flow cytometry
☒	☐	MRI-based neuroimaging

Antibodies

Antibodies used	Anti-H3K9me3, Active Motif, Cat# 39161, 1:100 dilution for IF;<br>Anti-H3K27me3, Diagenode, Cat# C15410195, 1:200 dilution for IF;<br>Anti-H3K14ac, Active Motif, Cat# 39599, 1:50 dilution for IF;<br>Anti-CBX1, Proteintech, Cat# 10241-2-AP, 1:50 dilution for IF;

Anti-H3.1/3.2, Cosmo Bio, Cat# CAC-CE-039B, 1:100 dilution for IF;
Anti-H3.1, Novus Biologicals, Cat# NBP2-75524, 1:1000 dilution for WB;
Anti-H3.3, Thermo Fisher Scientific, Cat# MA5-24667, 1:100 dilution for IF, 1:1000 dilution for WB;
Anti-TUBULIN, Proteintech, Cat# 66031-1-Ig, 1:2000 dilution for WB;
Anti-ATRX, Santa Cruz, Cat# sc-55584, 1:100 dilution for IF;
anti-SNRP70, Abcam, Cat# ab83306, 1:1000 dilution for WB;
anti-GAPDH, Proteintech, Cat# 10494-1-AP, 1:1000 dilution for WB;
Anti-GFP, Invitrogen, Cat# A11122, 2ug per reaction in 400ul reaction system for pulldown experiment;
Anti-rabbit-IgG, Sigma-Aldrich, Cat# 12-370, 2ug per reaction in 400ul reaction system for pulldown experiment;
Alexa Fluor® 594 Donkey Anti-Rabbit IgG (H+L) Antibody, Fisher Scientific, Cat# A-21207, 1:500 dilution for IF;
Alexa Fluor® 594 Donkey Anti-Mouse IgG (H+L) Antibody, Fisher Scientific, Cat# A-21203, 1:500 dilution for IF;
Amersham ECL Rabbit IgG, HRP-linked whole Ab (from donkey), Cytiva, Cat# NA934, 1:10000 dilution for WB;
Amersham ECL Mouse IgG, HRP-linked whole Ab (from sheep), Cytiva, Cat# NA931, 1:10000 dilution for WB.

Validation

- Anti-H3K9me3, Active Motif, Cat# 39161, 1:100 dilution for IF: The antibody guarantee covers the use of the antibody for IF applications. The antibody has been referenced in 57 publications. https://www.activemotif.com/catalog/details/39161/histone-h3-trimethyl-lys9-antibody-pab
-Anti-H3K27me3, Diagenode, Cat# C15410195, 1:200 dilution for IF: The antibody guarantee covers the use of the antibody for IF applications. The antibody has been referenced in 38 publications. https://www.diagenode.com/en/p/h3k27me3-polyclonal-antibody-premium-50-mg-27-ml
-Anti-H3K14ac, Active Motif, Cat# 39599, 1:50 dilution for IF: The antibody guarantee covers the use of the antibody for IF applications. The antibody has been referenced in 8 publications. https://www.activemotif.com/catalog/details/39599/histone-h3-acetyl-lys14-antibody-pab
-Anti-CBX1, Proteintech, Cat# 10241-2-AP, 1:50 dilution for IF: The antibody guarantee covers the use of the antibody for IF applications. The antibody has been referenced in 6 publications. https://www.ptglab.com/products/CBX1-Antibody-10241-2-AP.htm
-Anti-H3.1/3.2, Cosmo Bio, Cat# CAC-CE-039B, 1:100 dilution for IF: The antibody guarantee covers the use of the antibody for IF applications. The antibody has been referenced in 2 publications. https://www.cosmobiousa.com/products/anti-histone-h3-1-hist1h3a-h3-2-hist1h3b-mab-clone-6g3c7
-Anti-H3.1, Novus Biologicals, Cat# NBP2-75524, 1:1000 dilution for WB: The antibody guarantee covers the use of the antibody for WB applications. The antibody has been referenced in 1 publication. https://www.novusbio.com/products/histone-h31-antibody_nbp2-75524
-Anti-H3.3, Thermo Fisher Scientific, Cat# MA5-24667, 1:100 dilution for IF, 1:1000 dilution for WB: The antibody guarantee covers the use of the antibody for WB applications. The antibody has been referenced in 1 publication. https://www.thermofisher.cn/cn/zh/antibody/product/Histone-H3-3-Antibody-clone-RM190-Recombinant-Monoclonal/MA5-24667
-Anti-TUBULIN, Proteintech, Cat# 66031-1-Ig, 1:2000 dilution for WB: The antibody guarantee covers the use of the antibody for WB applications. The antibody has been referenced in 600 publications. https://www.ptglab.com/products/tubulin-Alpha-Antibody-66031-1-Ig.htm
-Anti-ATRX, Santa Cruz, Cat# sc-55584, 1:100 dilution for IF: The antibody guarantee covers the use of the antibody for IF applications. The antibody has been referenced in 37 publications. https://www.scbt.com/p/atrx-antibody-d-5?productCanUrl=atrx-antibody-d-5&_requestid=1959733
-anti-SNRP70, Abcam, Cat# ab83306, 1:1000 dilution for WB: The antibody guarantee covers the use of the antibody for WB applications. The antibody has been referenced in 15 publications. https://www.abcam.cn/snrp70u1-70k-antibody-ab83306.html
-anti-GAPDH, Proteintech, Cat# 10494-1-AP, 1:1000 dilution for WB: The antibody guarantee covers the use of the antibody for WB applications. The antibody has been referenced in 4143 publications. https://www.ptglab.com/products/GAPDH-Antibody-10494-1-AP.htm
-Anti-GFP, Invitrogen, Cat# A11122, 2ug per reaction in 400ul reaction system for pulldown experiment: The antibody guarantee covers the use of the antibody for IP applications. The antibody has been referenced in 47 publications. https://www.thermofisher.cn/cn/zh/antibody/product/GFP-Tag-Antibody-11E5-Monoclonal/A-11121
-Anti-rabbit-IgG, Sigma-Aldrich, Cat# 12-370, 2ug per reaction in 400ul reaction system for pulldown experiment: The antibody guarantee covers the use of the antibody for IP applications. The antibody has been referenced in 357 publications. https://www.sigmaaldrich.cn/CN/zh/product/mm/12370
- Alexa Fluor® 594 Donkey Anti-Rabbit IgG (H+L) Antibody, Fisher Scientific, Cat# A-21207, 1:500 dilution for IF: The antibody guarantee covers the use of the antibody for IF applications. The antibody has been referenced in 1606 publications. https://www.thermofisher.com/antibody/product/Donkey-anti-Rabbit-IgG-H-L-Highly-Cross-Adsorbed-Secondary-Antibody-Polyclonal/A-21207
- Alexa Fluor® 594 Donkey Anti-Mouse IgG (H+L) Antibody, Fisher Scientific, Cat# A-21203, 1:500 dilution for IF: The antibody guarantee covers the use of the antibody for IF applications. The antibody has been referenced in 1008 publications. https://www.thermofisher.com/antibody/product/Donkey-anti-Mouse-IgG-H-L-Highly-Cross-Adsorbed-Secondary-Antibody-Polyclonal/A-21203
-Amersham ECL Rabbit IgG, HRP-linked whole Ab (from donkey), Cytiva, Cat# NA934, 1:10000 dilution for WB: The antibody guarantee covers the use of the antibody for WB applications as secondary antibody. https://www.cytivalifesciences.com.cn/zh/cn/shop/protein-analysis/blotting-and-detection/blotting-standards-and-reagents/amersham-ecl-hrp-conjugated-antibodies-p-06260
-Amersham ECL Mouse IgG, HRP-linked whole Ab (from sheep), Cytiva, Cat# NA931, 1:10000 dilution for WB: The antibody guarantee covers the use of the antibody for WB applications as secondary antibody. https://www.cytivalifesciences.com.cn/zh/cn/shop/protein-analysis/blotting-and-detection/blotting-standards-and-reagents/amersham-ecl-hrp-conjugated-antibodies-p-06260

Eukaryotic cell lines

Policy information about cell lines

Cell line source(s)

Human HEK293T cell line was purchased from ATCC (American Type Culture Collection) with catalog number CRL-3216. MEF cells were established from embryonic day 12.5-13.5 (E12.5-E13.5) in our laboratory.

Authentication

HEK293T and MEF cell lines were grown in DMEM (Gibco) supplemented with 10% FBS (Gibco) and 1 mM L-glutamine (Merk Millipore) at 37 °C in a humidified 5% CO2 incubator. The morphology was check the same as it described by ATCC.

Mycoplasma contamination	All cell lines were tested negative for mycoplasma contamination. None of the cell lines were contaminated.
Commonly misidentified lines (See ICLAC register)	No commonly misidentified lines were used.

Animals and other organisms

Policy information about studies involving animals; ARRIVE guidelines recommended for reporting animal research

Laboratory animals	Specific pathogen-free (SPF)-grade C57BL/6J, and DBA2 mice were purchased from Beijing Vital River Laboratory Animal Technology Co., Ltd. BDF1 hybrid mice were obtained by mating female C57BL/6J mice with male DBA2 mice. C57BL/6J, DBA2 and BDF1 mice, were housed in the animal facility at Tongji University, Shanghai, China. 6–8 weeks old female BDF1 mice were used for the experiment. All the mice had free access to food and water, and were housed in 12 hours light/12 hours dark cycle 22.1–22.3 °C and 33–44% humidity.
Wild animals	No wild animals were used.
Field-collected samples	No field-collected samples were used.
Ethics oversight	All experiments were performed in accordance with the University of Health Guide for the Care and Use of Laboratory Animals and were approved by the Biological Research Ethics Committee of Tongji University.

Note that full information on the approval of the study protocol must also be provided in the manuscript.
